# Supplementary material for: Impact of having a child on physical activity in the UK: a scoping review protocol
Source: BMJ Open. 2022 Sep 22;12(9):e063410. doi: 10.1136/bmjopen-2022-063410 (PMC9511554; doi:10.1136/bmjopen-2022-063410)
Supplement: Supplementary data [file bmjopen-2022-063410supp002.pdf]

SUPPLEMENTARY MATERIAL 2: SEARCH STRATEGIES

PUBLISHED LITERATURE SEARCH STRATEGIES

CINAHL (EBSCOhost)

In title:

(“Physical activity” OR Exercise OR “Sedentary behaviour”) AND (“Having a child” OR “Having children” OR Childbearing OR Preconception OR Pregnancy OR Postpartum OR Post-partum OR Parenthood OR Motherhood OR Fatherhood) AND (England OR “Northern Ireland” OR Scotland OR Wales OR “United Kingdom” OR UK)

In abstract:

(“Physical activity” OR Exercise OR “Sedentary behaviour”) AND (“Having a child” OR “Having children” OR Childbearing OR Preconception OR Pregnancy OR Postpartum OR Post-partum OR Parenthood OR Motherhood OR Fatherhood) AND (England OR “Northern Ireland” OR Scotland OR Wales OR “United Kingdom” OR UK)

Embase (Embase 1974 to 2022 February 04)

1. Physical activity.ti,ab.
2. Physical activity/
3. Exercise.ti,ab.
4. Exercise/
5. Sedentary behaviour.ti,ab.
6. Having a child.ti,ab.
7. Having children.ti,ab.
8. Childbearing.ti,ab.
9. Preconception.ti,ab.
10. Pregnancy.ti,ab.
11. Pregnancy/
12. Postpartum.ti,ab.
13. Post-partum.ti,ab.
14. Parenthood.ti,ab.
15. Parenthood/
16. Motherhood.ti,ab.
17. Fatherhood.ti,ab.
18. England.ti,ab.
19. England/
20. Northern Ireland.ti,ab.
21. Northern Ireland/
22. Scotland.ti,ab.
23. Scotland/
24. Wales.ti,ab.
25. Wales/
26. United Kingdom.ti,ab.
27. United Kingdom/
28. UK.ti,ab.
29. 1 OR 2 OR 4 OR 4 OR 5
30. 6 OR 7 OR 8 OR 9 OR 10 OR 11 OR 12 OR 13 OR 14 OR 15 OR 16 OR 17
31. 18 OR 19 OR 20 OR 21 OR 22 OR 23 OR 24 OR 25 OR 26 OR 27 OR 28
32. 29 AND 30 AND 31

Medline (Ovid MEDLINE® 1946 to present)

1. Physical activity.ti,ab.
2. Exercise.ti,ab.
3. Exercise/
4. Sedentary behaviour.ti,ab.
5. Sedentary behaviour/
6. Having a child.ti,ab.
7. Having children.ti,ab.
8. Childbearing.ti,ab.
9. Preconception.ti,ab.
10. Pregnancy.ti,ab.
11. Pregnancy/
12. Postpartum.ti,ab.
13. Post-partum.ti,ab.
14. Parenthood.ti,ab.
15. Motherhood.ti,ab.
16. Fatherhood.ti,ab.
17. England.ti,ab.
18. England/
19. Northern Ireland.ti,ab.
20. Northern Ireland/
21. Scotland.ti,ab.
22. Scotland/
23. Wales.ti,ab.
24. Wales/
25. United Kingdom.ti,ab.
26. United Kingdom/
27. UK.ti,ab.
28. 1 OR 2 OR 3 OR O4 OR 5
29. 6 OR 7 OR 8 OR 9 OR 10 OR 11 OR 12 OR 13 OR 14 OR 15 OR 16
30. 17 OR 18 OR 19 OR 20 OR 21 OR 22 OR 23 OR 24 OR 25 OR 26 OR 27
31. 28 AND 29 AND 30

PsycInfo (APA PsychInfo 1806 to January Week 5 2022)

1. Physical activity.ti,ab.
2. Physical activity/
3. Exercise.ti,ab.
4. Exercise/
5. Sedentary behaviour.ti,ab.
6. Sedentary behaviour/
7. Having a child.ti,ab.
8. Having children.ti,ab.
9. Childbearing.ti,ab.
10. Preconception.ti,ab.
11. Pregnancy.ti,ab.
12. Pregnancy/
13. Postpartum.ti,ab.
14. Post-partum.ti,ab.
15. Parenthood.ti,ab.
16. Motherhood.ti,ab.
17. Fatherhood.ti,ab.
18. England.ti,ab.

19. Northern Ireland.ti,ab.
20. Scotland.ti,ab.
21. Wales.ti,ab.
22. United Kingdom.ti,ab.
23. UK.ti,ab.
24. 1 or 2 or 3 or 4 or 5 or 6
25. 7 or 8 or 9 or 10 or 11 or 12 or 13 or 14
26. 15 or 16 or 17 or 18 or 19 or 20
27. 21 and 22 and 23

Web of Science (Web of Science Core Collection)
In title:

((TI=("physical activity" OR exercise OR “sedentary behaviour”)) AND TI=("having a child" OR "having children" OR childbearing OR preconception OR pregnancy OR postpartum OR post-partum OR parenthood OR motherhood OR fatherhood)) AND TI=(england OR "northern ireland" OR scotland OR wales OR "united kingdom" OR uk)

In abstract:

((AB=("physical activity" OR exercise OR “sedentary behaviour”)) AND AB=("having a child" OR "having children" OR childbearing OR preconception OR pregnancy OR postpartum OR post-partum OR parenthood OR motherhood OR fatherhood)) AND AB=(england OR "northern ireland" OR scotland OR wales OR "united kingdom" OR uk)

GREY LITERATURE SEARCH STRATEGIES

Google Scholar
allintitle: ("physical activity" OR exercise OR “sedentary behaviour”) AND ("having a child" OR "having children" OR childbearing OR preconception OR pregnancy OR postpartum OR post-partum OR parenthood OR motherhood OR fatherhood) AND england

allintitle:("physical activity" OR exercise OR “sedentary behaviour”), ("having a child" OR "having children" OR childbearing OR preconception OR pregnancy OR postpartum OR post-partum OR parenthood OR motherhood OR fatherhood) AND "northern ireland"

allintitle: ("physical activity" OR exercise OR “sedentary behaviour”) AND ("having a child" OR "having children" OR childbearing OR preconception OR pregnancy OR postpartum OR post-partum OR parenthood OR motherhood OR fatherhood) AND Scotland

allintitle: ("physical activity" OR exercise OR “sedentary behaviour”) AND ("having a child" OR "having children" OR childbearing OR preconception OR pregnancy OR postpartum OR post-partum OR parenthood OR motherhood OR fatherhood) AND wales

allintitle: ("physical activity" OR exercise OR “sedentary behaviour”) AND ("having a child" OR "having children" OR childbearing OR preconception OR pregnancy OR postpartum OR post-partum OR parenthood OR motherhood OR fatherhood) AND "united kingdom"

allintitle: ("physical activity" OR exercise OR “sedentary behaviour”) AND ("having a child" OR "having children" OR childbearing OR preconception OR pregnancy OR postpartum OR post-partum OR parenthood OR motherhood OR fatherhood) AND uk

ProQuest
In title:

ti("physical activity" OR exercise OR “sedentary behaviour”) AND ti("having a child" OR "having children" OR childbearing OR preconception OR pregnancy OR postpartum OR post-partum OR

parenthood OR motherhood OR fatherhood) AND ti(england OR "northern ireland" OR scotland OR wales OR "united kingdom" OR uk)

In abstract:

ab("physical activity" OR exercise OR “sedentary behaviour”) AND ab("having a child" OR "having children" OR childbearing OR preconception OR pregnancy OR postpartum OR post-partum OR parenthood OR motherhood OR fatherhood) AND ab(england OR "northern ireland" OR scotland OR wales OR "united kingdom" OR uk)

Websites
For example, “Best Beginning’s” and “Tommy’s”.
