# Supplementary material for: Impact of having a child on physical activity in the UK: a scoping review protocol
Source: BMJ Open. 2022 Sep 22;12(9):e063410. doi: 10.1136/bmjopen-2022-063410 (PMC9511554; doi:10.1136/bmjopen-2022-063410)
Supplement: Author's manuscript [file bmjopen-2022-063410.draft_revisions.pdf]

BMJ Open is committed to open peer review. As part of this commitment we make the peer review history of every article we publish publicly available.

When an article is published we post the peer reviewers' comments and the authors' responses online. We also post the versions of the paper that were used during peer review. These are the versions that the peer review comments apply to.

The versions of the paper that follow are the versions that were submitted during the peer review process. They are not the versions of record or the final published versions. They should not be cited or distributed as the published version of this manuscript.

BMJ Open is an open access journal and the full, final, typeset and author-corrected version of record of the manuscript is available on our site with no access controls, subscription charges or pay-per-view fees (http://bmjopen.bmj.com).

If you have any questions on BMJ Open's open peer review process please email info.bmjopen@bmj.com

BMJ Open

The impact of having a child on physical activity in the United Kingdom: A scoping review protocol

Journal:	*BMJ Open*
Manuscript ID	bmjopen-2022-063410
Article Type:	Protocol
Date Submitted by the Author:	30-Mar-2022
Complete List of Authors:	Northcote, Matthew; University of Bristol, School for Policy Studies<br>Foster, Charlie ; University of Bristol, School for Policy Studies<br>Pulsford, Richard; University of Exeter, College of Life and Environmental Sciences<br>Spotswood, Fiona; University of Bristol, School of Management
Keywords:	PUBLIC HEALTH, SOCIAL MEDICINE, SPORTS MEDICINE

SCHOLARONE™
Manuscripts

The impact of having a child on physical activity in the United Kingdom: A scoping review protocol

Matthew Northcote[1], Charlie Foster[1], Richard Pulsford[2], Fiona Spotswood[3]

[1]School for Policy Studies, University of Bristol, Bristol, United Kingdom; [2]College of Life and Environmental Sciences, University of Exeter, Exeter, United Kingdom; [3]School of Management, University of Bristol, Bristol, United Kingdom

Word count: 2311 (excluding title page, abstract, strengths and limitations of this study, keywords, acknowledgements, references, contributorship statement, funding statement, and competing interests statement)

Corresponding author: Matthew Northcote (12 Woodland Road, City of Bristol, Bristol, BS8 1UQ; av20205@bristol.ac.uk)

For peer review only

TABLE OF CONTENTS

ABSTRACT 1

Introduction 1

Methods and analysis 1

Ethics and dissemination 1

Scoping review registration 1

STRENGTHS AND LIMITATIONS OF THIS STUDY 2

KEYWORDS 2

INTRODUCTION 2

METHODS 3

Patient and public involvement statement 4

Stage 1: Identify the research questions 4

Stage 2: Identifying relevant studies 5

Eligibility criteria 5

Search strategy 5

Stage 3: Study selection 7

Stage 4: Charting the data 7

Stage 5: Collating, summarising, and reporting the findings 8

Critical appraisal of individual sources of evidence 8

DISSEMINATION AND COMMUNICATION OF RESULTS 8

CONCLUSION 9

ACKNOWLEDGEMENTS 9

CONTRIBUTORSHIP STATEMENT 9

COMPETING INTERESTS 9

FUNDING 9

DATA SHARING STATEMENT 10

REFERENCES 10

For peer review only

ABSTRACT

Introduction

Throughout the life course, there are major life transitions that are associated with reduced physical activity, which may have further implications for health and wellbeing. Having a child is one such transition that has been identified as a critical transformative experience and inflection point for obesity. We will conduct a scoping review of available evidence exploring the impact of having a child on physical activity in the United Kingdom.

Methods and analysis

We will use best practice methodological frameworks to guide the conduct of this scoping review. This method will enable us to map key concepts and available evidence, summarise and disseminate findings to key stakeholders, identify knowledge gaps, and determine the main priorities for future research. A three-step search strategy will identify relevant primary research studies, including reviews, from published and grey literature. An initial limited search will identify pertinent reviews from which keywords and index terms will be extracted. We will then select search terms and conduct main searches. Two reviewers will independently screen titles and abstracts of identified studies for inclusion and chart data, with a third reviewer resolving any conflicts. Backwards citation tracking will identify any additional studies. We will conduct numerical and thematic analysis to map data in a tabular and diagrammatic format and provide a description of key concepts and findings by theme, respectively.

Ethics and dissemination

We will disseminate findings to key stakeholder groups, practitioners, and policymakers through peer-reviewed and non-peer-reviewed publications, conferences, social media platforms, and in-person communications. Consultations with key stakeholders and their unique expertise and perspectives will provide greater insight. We will establish the main priorities for future research to inform the aims and objectives of subsequent studies.

Scoping review registration

Open Science Framework https://osf.io/gtqa4/ DOI 10.17605/OSF.IO/GTQA4

For peer review only

For peer review only - http://bmjopen.bmj.com/site/about/guidelines.xhtml

STRENGTHS AND LIMITATIONS OF THIS STUDY

- This study will be the first to use a scoping review methodology to provide an overview of available evidence, including published and grey literature, exploring the impact of having a child on physical activity in the United Kingdom.
- Our review will attempt to answer broad research questions to map key concepts and available evidence, summarise and disseminate findings to key stakeholders, identify knowledge gaps, and determine the main priorities for future research.

For peer review only

KEYWORDS

Childbearing; England; Exercise; Fatherhood; Having a child; Having children; Motherhood; Northern Ireland; Parenthood; Physical activity; Postpartum; Post-partum; Preconception; Pregnancy; Scotland; Sedentary behaviour; UK; United Kingdom; Wales

INTRODUCTION

Physical activity (PA) can be defined as “any bodily movement produced by the skeletal muscles that results in energy expenditure” [1]. The evidence for PA as a key component of physical, mental, and social health and wellbeing is well-established [2]. Despite this, recent evidence suggests that over a third (38%) of adults in England are less aerobically active than current public health guidelines recommend [3]. In addition, around 35% of adults who met aerobic activity recommendations did not meet those for muscle-strengthening activity [3]. Physical inactivity accounts for one in six deaths in the United Kingdom (UK) (equivalent to smoking) and has an estimated annual cost of £7.4 billion, including £0.9 billion to the National Health Service (NHS) alone [4]. Given the prevalence and economic and social burden of physical inactivity, it is important to identify those within the UK who may be less physically active in order to characterise and understand the contexts and circumstances that foster inactivity across the life course.

Throughout the life course, there are major life transitions that are associated with reduced PA, which may have further implications for health and wellbeing [5]. Having a child is one such transition that has been identified as a critical transformative experience and inflection point for

For peer review only - http://bmjopen.bmj.com/site/about/guidelines.xhtml

obesity [6]. Moreover, having a child is a life transition which is often coupled with others, including changes to relationship status, and living situation [7]. Existing evidence suggests having a child reduces PA, but largely focusses on mothers across the preconception, pregnancy, and postpartum periods and into parenthood [8]. However, having a child is also a major life transition for other members of the family unit, such as fathers, where this population has been relatively under-researched [9]. In England, there were 585,195 live births in 2020 [10], where around 45% are unintended, which may further exacerbate this issue [11]. Consequently, it is important to promote PA among those having a child in the UK.

Preliminary searches of Medline and Google Scholar for relevant reviews broadened knowledge of available evidence and informed the formulation of research questions. There were no reviews exploring the impact of having a child on PA in the UK. Further preliminary searches for relevant primary research studies identified only one study which examined the association between family-related life events (i.e., cohabitation/marriage and becoming a parent) and change in PA in the UK [12]. Findings from this study suggest that becoming a parent was associated with a decline in PA among men (β = -0.234, 95% CI -0.396 to -0.072), particularly non-cohabiting fathers (β = -0.937, 95% CI -1.623 to -0.250), but not women (β = 0.126, 95% CI -0.048 to 0.301). Since no relevant reviews were identified and a dearth of primary research studies, we aim to provide an overview of available evidence exploring the impact of having a child on PA in the UK.

METHODS

We will conduct a study exploring the impact of having a child on PA in the UK using a scoping review methodology. Although there is no consensus definition for scoping reviews [13-14], it is a form of review which can map key concepts and available evidence within a given topic area, exploring the extent, range, and nature of existing evidence [15-16]. Moreover, scoping reviews can define and clarify existing concepts, summarise and disseminate findings to key stakeholders, such as policymakers and practitioners, identify knowledge gaps, and determine the main priorities for future research [15-16].

A scoping review, rather than a systematic review, is more congruent with the aims and purposes of this study. Specifically, scoping reviews use broad research questions to provide an overview of the

For peer review only - http://bmjopen.bmj.com/site/about/guidelines.xhtml

For peer review only

available evidence [15]. Meanwhile, systematic reviews use focussed research questions to identify the best evidence. Despite being useful as a precursor to systematic reviews, scoping reviews are legitimate in their own right as standalone studies and are not required to be followed up with a systematic review [15]. Furthermore, although scoping reviews are not, by name, systematic, they do follow a rigorous systematic process [17-18].

We will use best-practice scoping review methodological frameworks by Arksey and O'Malley [15], adapted by Levac et al. [16] and the Joanna Briggs Institute (JBI) [18-19], to guide the conduct of this study. This is an iterative process by which research questions and methods may be further developed with the emergent evidence. We will report any deviation from this protocol in the final review. In addition, we will use the Preferred Reporting Items for Systematic reviews and Meta-Analysis extension for Scoping Reviews (PRISMA-ScR) to guide the reporting of this protocol as detailed in **online supplementary material 1** [20]. Furthermore, methods were informed by previous scoping reviews in PA for health research [21-23].

Patient and public involvement statement

Patients and members of the public were not involved in the preparation of this protocol.

Stage 1: Identify the research questions

The following broad research questions were informed by preliminary searches and interdisciplinary discussions within the research team. Moreover, research questions were formulated using the P.C.C. (Population, Concept, Context) mnemonic which will help to identify relevant studies and establish appropriate eligibility criteria [19].

- What is known about the impact of having a child on PA in the UK?
- What are the knowledge gaps within this field?
- What are the main priorities for future research in this field?

For peer review only - http://bmjopen.bmj.com/site/about/guidelines.xhtml

Stage 2: Identifying relevant studies

Eligibility criteria

The following a priori eligibility criteria was informed by preliminary searches, interdisciplinary discussions within the research team, and research questions.

- Participants have to be adults that were planning to have (i.e., preconception to pregnancy), having (i.e., throughout pregnancy), have had (i.e., postpartum and into parenthood), or planned to have (i.e., stillbirth) a child.
- Participants can be from any group, including general and specific populations (e.g., those recruited based on their living with a physical or mental illness or condition).
- Participants can be of any sex, age group, and ethnicity.
- Findings must explore the impact of having a child on PA, but not the impact of PA on health and wellbeing.
- Findings must report data on PA and/or sedentary behaviour which was assessed either quantitatively (i.e., device-derived or through self-report) and/or qualitatively (e.g., attitudes, views, perspectives, experiences, etc.).
- Studies can be published literature (e.g., primary research studies, including reviews) or grey literature (e.g., unpublished dissertations/theses or conference proceedings).
- Studies must be of quantitative observational (e.g., cross-sectional, prospective cohort), qualitative, or mixed methods study design.
- Studies must not have an experimental study design (e.g., randomised controlled trials [RCTs]) evaluating adherence and/or acceptability of interventions, or secondary analysis thereof; guidelines, opinion pieces (e.g., commentaries or editorials); or popular press articles (e.g., magazine or newspaper articles).
- Studies must be conducted with data generated exclusively within the UK, including England, Northern Ireland, Scotland, and Wales.
- Studies must be written in the English language.

For peer review only

Search strategy

Step 1: An initial limited search

We conducted an initial limited search of Medline (Ovid) and Google Scholar (Advanced Search) for relevant reviews. Given that there were no reviews exploring the impact of having a child on PA in the UK, we expanded search terms to allow for those using data generated elsewhere, including

For peer review only - http://bmjopen.bmj.com/site/about/guidelines.xhtml

"having a child" AND "physical activity" AND "review". The two records identified from Medline and the first 100 of the 6,720 records identified from Google Scholar were reviewed. A total of five reviews were identified as relevant. We also conducted a search of ProQuest to identify any similar dissertations but did not retrieve any records.

Step 2: Identify keywords and search terms

We used keywords and index terms featured within the titles and abstracts of the relevant reviews to inform main searches. An a priori search strategy for published literature within the Medline electronic bibliographic database, with Boolean operators and Medical Subject Headings (i.e., MeSH terms) is detailed in **online supplementary material 2**. We will adapt this search strategy for searches within CINAHL (EBSCOhost), Embase (Ovid), PsycINFO (Ovid), and Web of Science databases (Clarivate). Search terms will relate to those life stages and transitions involved in "having a child" in an attempt to remain inclusive, where having a child can impact a variety of populations. Moreover, search terms will relate to the concept of "physical activity" and the context of the "United Kingdom". We have made a pragmatic decision to only search for studies which have these search terms in the title and/or abstract due to the practical constraints of this study.

We will use a similar search strategy for grey literature, where the search terms used for Medline will be adapted for Google Scholar and ProQuest. In Google Scholar, this will involve a reduced set of search terms and multiple searches due to the limitation on characters. Moreover, in Google Scholar, it is only possible to search for studies which have these search terms in the title. We will also search websites of organisations (e.g., Best Beginning's, Tommy's) involved in the commissioning, undertaking, or cataloguing of research in this field and, if appropriate, we will make contact by email.

Step 3: Further searching of references and citations

We will conduct backwards citation tracking of included studies to identify further potentially eligible studies. Considering that scoping reviews are typically iterative, additional search terms and information sources (e.g., electronic bibliographic databases and websites) may be added to existing search strategies at any time throughout the research process. We may also contact corresponding authors of included studies to request further information. After completion of this study, we will make search strategies available upon request from the corresponding author and as published

For peer review only - http://bmjopen.bmj.com/site/about/guidelines.xhtml

supplementary material. We will import references of identified studies into Endnote X9.2 reference management software and organise them by database of origin, with duplicates removed.

Stage 3: Study selection

Titles and abstracts of identified studies will be predominantly screened for eligibility by one researcher (MN). A second researcher (JB) will independently complete this same process on a random sample of 10% of titles and abstracts. Any conflicts will be resolved by a third researcher (CF/RP). If there is no consensus between these three parties, then we will include the study in the review. All researchers will meet to discuss the eligibility criteria before embarking on this process to minimise these occurrences. If the same screening decisions are taken by both researchers for 95% of studies, then MN will screen the remaining titles and abstracts. Meanwhile, if the same decisions are taken for less than 95% of studies, then MN and JB will meet to discuss these decisions with reference to the eligibility criteria and repeat this process.

Stage 4: Charting the data

Data charting of included studies will be predominantly conducted by MN. A data charting form will be employed, using the a priori headings detailed below [19]. Given that scoping reviews are typically iterative, additional headings may be added to the form throughout the research process. The data charting process will involve MN charting 90% of studies and JB charting 10% of studies, with JB checking 10% of MN’s data and vice versa. Any conflicts will be resolved by CF/RP during group meetings. If there are considerable differences between researchers, MN and JB will meet to discuss decisions with reference to the data charting form and rectify any issues. If a full text article cannot be retrieved, MN will attempt to gain access through the university library and by contacting corresponding authors. If the full text article is not accessible, the study will be excluded.

- Author(s).
- Year of publication.
- Source and country of origin
- Aims/purpose.
- Study population and sample size (if applicable).
- Methodology/methods
- Concept

For peer review only - http://bmjopen.bmj.com/site/about/guidelines.xhtml

- Findings and details of these (e.g., how measured) (if applicable).
- Key findings by theme that relate to research questions.

Stage 5: Collating, summarising, and reporting the findings

We will conduct a numeric analysis of included studies to describe review decisions, findings from searches, removal of duplicates, study selection, full text retrieval, and backwards citation tracking [19]. We will present these findings using a study flow diagram. In addition, we will map data in a tabular and diagrammatic format by period of publication, country of origin, study type, and themes. Considering that scoping reviews are typically an iterative process, we may include additional categories of data. We will also conduct a thematic analysis of included studies using NVivo 12 qualitative data analysis software to provide a description of key concepts relevant to research questions and findings by theme. Furthermore, we will use the PRISMA-ScR framework to guide the reporting of this study [20].

Critical appraisal of individual sources of evidence

We will not conduct critical appraisal of individual sources of evidence, where this is consistent with methodological frameworks for scoping reviews [15].

DISSEMINATION AND COMMUNICATION OF RESULTS

This study will provide a novel insight into the impact of having a child on PA in the UK. Specifically, we will map key concepts and available evidence, identify knowledge gaps, and determine the main priorities for future research. We will subsequently summarise and submit findings for peer review and publication in a relevant open-access journal. Moreover, we will develop a series of infographics to be disseminated at conferences and online via professional social media channels, such as Twitter. Importantly, findings from this study will be reported regardless of the nature of the relationships between having a child and PA in the UK. We also aim to consult with key stakeholders, including Bristol City Council, Sport England, and Public Health England, to comment on findings and provide greater insight with their unique expertise and perspectives [16,24].

For peer review only - http://bmjopen.bmj.com/site/about/guidelines.xhtml

CONCLUSION

This protocol outlines our study to explore the impact of having a child on PA in the UK. We will conduct a scoping review using best-practice methodological frameworks to map key concepts and available evidence, identify knowledge gaps, and determine the main priorities for future research. Findings will be summarised and disseminated to stakeholder groups, practitioners, and policymakers through peer-reviewed and non-peer-reviewed publications, conferences, social media platforms, and in-person communications.

ACKNOWLEDGEMENTS

The authors would like to thank Dr Joey Murphy for their guidance in identifying relevant literature pertaining to best-practice methodological frameworks.

For peer review only

CONTRIBUTORSHIP STATEMENT

MN, CF, RP, and FS formulated the research questions for this study. CF identified the methodological frameworks and methods. MN prepared the methods. MN, CF, and RP contributed to the development of the search strategy. Jack Brazier (JB), a non-author, accepted the role of second researcher within the study selection and data charting processes. MN wrote the first and subsequent drafts of the manuscript. All authors reviewed and approved the manuscript.

COMPETING INTERESTS

None declared.

FUNDING

This study was part of MN’s PhD research at the Centre for Exercise, Nutrition, and Health Sciences, School for Policy Studies, University of Bristol. MN is a PhD Researcher with the South West Doctoral Training Partnership (SWDTP) which is funded by the Economic and Social Research Council (ESRC).

For peer review only - http://bmjopen.bmj.com/site/about/guidelines.xhtml

DATA SHARING STATEMENT

Not applicable.

REFERENCES

1 Capsersen CJ, Powell KE, Christenson GM. Physical activity, exercise, and physical fitness: definitions and distinctions for health-related research. *Public Health Rep* 1985;100(2):126–130.

2 Physical Activity Guidelines Advisory Committee. Physical Activity Guidelines Advisory Committee Scientific Report. Washington, DC: United States Department of Health and Human Services 2018.

[dataset] [3] NHS Digital. Data from: Health Survey for England, 2016: Adult physical activity – tables. December 13, 2017.

4 Office for Health Improvement and Disparities. Physical activity: applying All Our Health. 2022.

5 Allender S, Hutchinson L, Foster C. Life-change events and participation in physical activity: a systematic review. *Health Promot Int* 2008;23(2):160–172 http://dx.doi.org/10.1093/heapro/dan012 [published Online First: 25 March 2008].

6 Saxbe D, Rossin-Slater M, Goldenberg D. The transition to parenthood as a critical window for adult health. *Am Psychol* 2018;73(9):1190–1200 http://dx.doi.org/10.1037/amp0000376.

7 Hull EE, Rofey DL, Robertson RJ, et al. Influence of marriage and parenthood on physical activity: a 2-year prospective analysis. *J Phys Act Health* 2010;7(5):577–583 http://dx.doi.org/10.1123/jpah.7.5.577.

For peer review only

8 Bellows-Riecken KH, Rhodes RE. A birth of inactivity? A review of physical activity and parenthood. *Prev Med* 2008;46(2):99–110 http://dx.doi.org/10.1016/j.ypmed.2007.08.003 [published Online First: 15 August 2007].

9 Pot N, Keizer R. Physical activity and sport participation: A systematic review of the impact of fatherhood. *Prev Med Rep* 2016;4:121–127 http://dx.doi.org/10.1016/j.pmedr.2016.05.018 [published Online First: 31 May 2016].

[dataset] [10] Office for National Statistics. Data from: Births in England and Wales: summary tables. October 14, 2021.

11 Wellings K, Jones KG, Mercer CH, et al. The prevalence of unplanned pregnancy and assicated factors in Britain: findings from the third National Survey of Sexual Attitudes and Lifestyles (Natsal-3). *Lancet* 2013;382(9907):1807–1816 https://doi.org/10.1016/S0140-6736(13)62071-1 [published Online First: 26 November 2013].

12 Werneck AO, Winpenny EM, Van Sluijs EM, et al. Cohabiting and becoming a parent: associations with changes in physical activity in the 1970 British cohort study. *BMC Public Health* 2020;20(1):1–9 http://dx.doi.org/10.1186/s12889-020-09187-2 [published Online First: 10 July 2020].

13 Colquhoun HL, Levac D, O'Brien KK, et al. Scoping reviews: time for clarity in definition, methods, and reporting. *J Clin Epidemiol* 2014;67(12):1291–1294 http://dx.doi.org/10.1016/j.jclinepi.2014.03.013 [published Online First: 14 July 2014].

14 Peterson J, Pearce PF, Ferguson LA, et al. Understanding scoping reviews: Definition, purpose, and process. *J Am Assoc Nurse Pract* 2017;29(1):12–16 http://dx.doi.org/10.1002/2327-6924.12380.

For peer review only

For peer review only - http://bmjopen.bmj.com/site/about/guidelines.xhtml

15 Arksey H, O'Malley L. Scoping studies: towards a methodological framework. *Int J Soc Res Methodol* 2005;8(1):19–32 https://doi.org/10.1080/1364557032000119616 [published Online First: 23 February 2007].

16 Levac D, Colquhoun H, O'Brien KK. Scoping studies advancing the methodology. *Implement Sci* 2010;5(1):1–9 http://dx.doi.org/10.1186/1748-5908-5-69 [published Online First: 20 September 2010].

17 Polit DF, Beck CT. Systematic reviews of research evidence: Meta-analysis, metasynthesis, and mixed studies review. In: Polit DF, Beck CT, 9th ed. Nursing research: Generating and assessing evidence for nursing practice. Philadeplhia, PA. 2012.

18 Peters MD, Marnie C, Tricco AC, et al. Updated methodological guidance for the conduct of scoping reviews. *JBI Evid Synth* 2020;18(10):2119–2126 http://dx.doi.org/10.11124/JBIES-20-00167.

19 Peters MD, Godfrey CM, Khalil H, et al. Guidance for conducting systematic scoping reviews. *Int J Evid Based Healthc* 2015;13(3):141–146 http://dx.doi.org/10.1097/XEB.0000000000000050.

20 Tricco AC, Lillie E, Zarin W, et al. PRISMA extension for scoping reviews (PRISMA-ScR): cheecklist and explanation. *Ann Intern Med* 2018;169(7):467–473 http://dx.doi.org/10.7326/M18-0850 [published Online First: 2 October 2018].

21 Murray A, Daines L, Archibald D, et al. The relationship and effects of golf on physical and mental health: a scoping review protocol. *Br J Sports Med* 2016;50(11):647–650 http://dx.doi.org/10.1136/bjsports-2015-095914 [published Online First: 29 April 2016].

22 Griffin SA, Perera NK, Murray A, et al. The relationships between rugby union and health: a scoping review protocol. *BMJ Open Sport Exerc Med* 2019;5(1):1–5 http://dx.doi.org/10.1136/bmjsem-2019-000593 [published Online First: 24 August 2019].

For peer review only

For peer review only - http://bmjopen.bmj.com/site/about/guidelines.xhtml

23 Gropper H, John JM, Sudeck G, et al. The impact of life events and transitions on physical activity: A scoping review. *PLoS One* 2020;15(6):1–24 http://dx.doi.org/10.1371/journal.pone.0234794 [published Online First: 22 June 2020].

24 Pham MT, Rajić A, Greig JD, et al. A scoping review of scoping reviews: advancing the approach and enhancing the consistency. *Res Synth Methods* 2014;5(4):371–85 http://dx.doi.org/10.1002/jrsm.1123 [published Online First: 24 July 2014].

For peer review only

For peer review only - http://bmjopen.bmj.com/site/about/guidelines.xhtml

Preferred Reporting Items for Systematic reviews and Meta-Analyses extension for Scoping Reviews (PRISMA-ScR) Checklist

SECTION	ITEM	PRISMA-ScR CHECKLIST ITEM	REPORTED ON PAGE #
**TITLE**			
Title	1	Identify the report as a scoping review.	Title page
**ABSTRACT**			
Structured summary	2	Provide a structured summary that includes (as applicable): background, objectives, eligibility criteria, sources of evidence, charting methods, results, and conclusions that relate to the review questions and objectives.	1
**INTRODUCTION**			
Rationale	3	Describe the rationale for the review in the context of what is already known. Explain why the review questions/objectives lend themselves to a scoping review approach.	2-3
Objectives	4	Provide an explicit statement of the questions and objectives being addressed with reference to their key elements (e.g., population or participants, concepts, and context) or other relevant key elements used to conceptualize the review questions and/or objectives.	3-4
**METHODS**			
Protocol and registration	5	Indicate whether a review protocol exists; state if and where it can be accessed (e.g., a Web address); and if available, provide registration information, including the registration number.	1
Eligibility criteria	6	Specify characteristics of the sources of evidence used as eligibility criteria (e.g., years considered, language, and publication status), and provide a rationale.	5
Information sources*	7	Describe all information sources in the search (e.g., databases with dates of coverage and contact with authors to identify additional sources), as well as the date the most recent search was executed.	5-6
Search	8	Present the full electronic search strategy for at least 1 database, including any limits used, such that it could be repeated.	6
Selection of sources of evidence†	9	State the process for selecting sources of evidence (i.e., screening and eligibility) included in the scoping review.	7
Data charting process‡	10	Describe the methods of charting data from the included sources of evidence (e.g., calibrated forms or forms that have been tested by the team before their use, and whether data charting was done independently or in duplicate) and any processes for obtaining and confirming data from investigators.	7
Data items	11	List and define all variables for which data were sought and any assumptions and simplifications made.	5, 7-8
Critical appraisal of individual sources of evidence§	12	If done, provide a rationale for conducting a critical appraisal of included sources of evidence; describe the methods used and how this information was used in any data synthesis (if appropriate).	NA

St. Michael's
Inspired Care.
Inspiring Science.
For peer review only - http://bmjopen.bmj.com/site/about/guidelines.xhtml

SECTION	ITEM	PRISMA-ScR CHECKLIST ITEM	REPORTED ON PAGE #
Synthesis of results	13	Describe the methods of handling and summarizing the data that were charted.	8
**RESULTS**			
Selection of sources of evidence	14	Give numbers of sources of evidence screened, assessed for eligibility, and included in the review, with reasons for exclusions at each stage, ideally using a flow diagram.	NA
Characteristics of sources of evidence	15	For each source of evidence, present characteristics for which data were charted and provide the citations.	NA
Critical appraisal within sources of evidence	16	If done, present data on critical appraisal of included sources of evidence (see item 12).	NA
Results of individual sources of evidence	17	For each included source of evidence, present the relevant data that were charted that relate to the review questions and objectives.	NA
Synthesis of results	18	Summarize and/or present the charting results as they relate to the review questions and objectives.	NA
**DISCUSSION**			
Summary of evidence	19	Summarize the main results (including an overview of concepts, themes, and types of evidence available), link to the review questions and objectives, and consider the relevance to key groups.	NA
Limitations	20	Discuss the limitations of the scoping review process.	3-4
Conclusions	21	Provide a general interpretation of the results with respect to the review questions and objectives, as well as potential implications and/or next steps.	NA
**FUNDING**			
Funding	22	Describe sources of funding for the included sources of evidence, as well as sources of funding for the scoping review. Describe the role of the funders of the scoping review.	12

JBI = Joanna Briggs Institute; PRISMA-ScR = Preferred Reporting Items for Systematic reviews and Meta-Analyses extension for Scoping Reviews.
* Where *sources of evidence* (see second footnote) are compiled from, such as bibliographic databases, social media platforms, and Web sites.
† A more inclusive/heterogeneous term used to account for the different types of evidence or data sources (e.g., quantitative and/or qualitative research, expert opinion, and policy documents) that may be eligible in a scoping review as opposed to only studies. This is not to be confused with *information sources* (see first footnote).
‡ The frameworks by Arksey and O'Malley (6) and Levac and colleagues (7) and the JBI guidance (4, 5) refer to the process of data extraction in a scoping review as data charting.
§ The process of systematically examining research evidence to assess its validity, results, and relevance before using it to inform a decision. This term is used for items 12 and 19 instead of "risk of bias" (which is more applicable to systematic reviews of interventions) to include and acknowledge the various sources of evidence that may be used in a scoping review (e.g., quantitative and/or qualitative research, expert opinion, and policy document).

From: Tricco AC, Lillie E, Zarin W, O'Brien KK, Colquhoun H, Levac D, et al. PRISMA Extension for Scoping Reviews (PRISMAScR): Checklist and Explanation. Ann Intern Med. 2018;169:467–473. doi: 10.7326/M18-0850.

For peer review only - http://bmjopen.bmj.com/site/about/guidelines.xhtml

1. physical activity.ti,ab.
2. physical activity/
3. exercise.ti,ab.
4. exercise/
5. sedentary behaviour.ti,ab.
6. having a child.ti,ab.
7. having children.ti,ab.
8. childbearing.ti,ab.
9. preconception.ti,ab.
10. pregnancy.ti,ab.
11. pregnancy/
12. postpartum.ti,ab.
13. post-partum.ti,ab.
14. parenthood.ti,ab.
15. parenthood/
16. motherhood.ti,ab.
17. fatherhood.ti,ab.
18. england.ti,ab.
19. England/
20. northern ireland.ti,ab.
21. Northern Ireland/
22. scotland.ti,ab.
23. Scotland/
24. wales.ti,ab.
25. Wales/
26. united kingdom.ti,ab.
27. United Kingdom/
28. uk.ti,ab.
29. 1 or 2 or 3 or 4 or 5
30. 6 or 7 or 8 or 9 or 10 or 11 or 12 or 13 or 14 or 15 or 16 or 17
31. 18 or 19 or 20 or 21 or 22 or 23 or 24 or 25 or 26 or 27 or 28
32. 29 and 30 and 31

For peer review only

BMJ Open

The impact of having a child on physical activity in the United Kingdom: A scoping review protocol

Journal:	*BMJ Open*
Manuscript ID	bmjopen-2022-063410.R1
Article Type:	Protocol
Date Submitted by the Author:	03-Aug-2022
Complete List of Authors:	Northcote, Matthew; University of Bristol, School for Policy Studies<br>Foster, Charlie ; University of Bristol, School for Policy Studies<br>Pulsford, Richard; University of Exeter, College of Life and Environmental Sciences<br>Spotswood, Fiona; University of Bristol, School of Management
**Primary Subject Heading**:	Public health
Secondary Subject Heading:	Health policy
Keywords:	PUBLIC HEALTH, SPORTS MEDICINE, SOCIAL MEDICINE

SCHOLARONE™
Manuscripts

THE IMPACT OF HAVING A CHILD ON PHYSICAL ACTIVITY IN THE UNITED KINGDOM: A SCOPING REVIEW PROTOCOL

Matthew Northcote[1], Charlie Foster[1], Richard Pulsford[2], Fiona Spotswood[3]

[1]School for Policy Studies, University of Bristol, Bristol, United Kingdom; [2]College of Life and Environmental Sciences, University of Exeter, Exeter, United Kingdom; [3]School of Management, University of Bristol, Bristol, United Kingdom

Word count: 3,723 (excluding title page, abstract, strengths and limitations of this study, keywords, acknowledgements, contributors, competing interests, funding, and references)

Corresponding author: Matthew Northcote (12 Woodland Road, City of Bristol, Bristol, BS8 1UQ; av20205@bristol.ac.uk)

For peer review only

ABSTRACT

Introduction

Throughout the life course, there are major life transitions that are associated with reduced physical activity, which may have further implications for health and wellbeing. Having a child is one such transition that has been identified as a critical transformative experience and window for intervention. We will conduct a scoping review of available evidence exploring the impact of having a child on physical activity in the United Kingdom.

Methods and analysis

We will use best-practice methodological frameworks to map key concepts and available evidence, summarise and disseminate findings to stakeholders, and identify knowledge gaps. A three-step search strategy will identify primary research studies, including reviews, from published and grey literature, exploring the impact of having a child on physical activity in the United Kingdom, from the preconception period, throughout pregnancy, the postpartum period, and into parenthood. An initial limited search will identify relevant reviews, from which keywords and index terms will be extracted. We will conduct searches of CINAHL, Embase, Medline, PsycINFO, and Web of Science to identify relevant articles written in English from inception to February 2022. Two reviewers will independently screen titles and abstracts of identified studies for inclusion and chart data, with a third reviewer resolving any conflicts. Backwards citation tracking will identify any additional studies. We will conduct numerical and thematic analysis to map data in tabular and diagrammatic format and provide a description of findings by theme.

Ethics and dissemination

Ethical approval is not required for this scoping review. We will disseminate findings to stakeholders through publications, conferences, social media platforms, and in-person communications. Consultations with key stakeholders, with their unique expertise and perspectives, will provide greater insight. We will establish the main priorities for future research to inform the research questions of subsequent studies.

Scoping review registration

Open Science Framework https://osf.io/gtqa4/ DOI 10.17605/OSF.IO/GTQA4

STRENGTHS AND LIMITATIONS OF THIS STUDY

- This study will use a scoping review methodology which can provide an overview of available evidence within a given area of research.
- Scoping reviews are a form of literature review which can map key concepts and available evidence, exploring the extent, range, and nature of existing evidence.
- These methods use broad research questions to define and clarify existing concepts, summarise and disseminate findings to key stakeholders, identify knowledge gaps, and determine the main priorities for future research.
- There are best-practice methodological frameworks available to guide the conduct of scoping reviews and established guidelines to inform the reporting of such studies.
- Scoping reviews do not address more focussed research questions, like in systematic reviews, nor do they generally include formal critical appraisal of individual sources of evidence using tools.

KEYWORDS

Childbearing; England; Exercise; Fatherhood; Having a child; Having children; Motherhood; Northern Ireland; Parenthood; Physical activity; Postpartum; Post-partum; Preconception; Pregnancy; Scotland; Sedentary behaviour; UK; United Kingdom; Wales

For peer review only - http://bmjopen.bmj.com/site/about/guidelines.xhtml

INTRODUCTION

Physical activity (PA) can be defined as 'any bodily movement produced by skeletal muscles that results in energy expenditure' [1]. The benefits of regular PA participation as a key component of physical, mental, and social health and wellbeing has been well-established [2]. For example, PA can reduce the risk of disease, manage existing conditions, and develop and maintain physical, mental, and social function [3].

Recent evidence from national health surveys in the United Kingdom (UK) indicate that a large proportion of adults (aged 16 and over) are less physically active than the current UK Chief Medical Officer's PA Guidelines recommend [3]. Specifically, 34% in England in 2016 [4]; 45% in Northern Ireland between 2016 and 2017 [5]; 54% in Scotland in 2020 [6]; and 44% in Wales between 2020 and 2021 [7] did not meet PA guidelines for aerobic activity (i.e., at least 150 minutes of moderate-intensity activity, or 75 minutes of vigorous-intensity activity, or shorter durations of very-vigorous-intensity activity, or combination of moderate-, vigorous-, and very-vigorous-intensity activity each week). It is necessary here to note the likely negative impact of lockdown measures to curb the spread of the 2019 coronavirus in surveys conducted between 2020 and 2021. Moreover, of those who met recommendations for aerobic activity, over a third, 35% in England in 2016 [4] and 37% in Scotland in 2019 [8], did not meet that for muscle-strengthening activity (i.e., at least two or more days each week).

According to the PA guidelines, adults are to minimise the amount of time spent being sedentary and break up long periods of inactivity with at least light-intensity activity [3]. Sedentary behaviour (SB) can be defined as 'any waking behaviour characterized by an energy expenditure ≤1.5 [metabolic equivalents of task (METs)] while in a sitting or reclining posture' [9]. In adults, high volumes of SB heighten the risk of all-cause mortality, cardiovascular mortality, cardiovascular disease incidence, and type-2 diabetes incidence [2,10-12]. In England, men are more likely than women to have increased time spent being sedentary during both the weekday (4.9 hours and 4.7 hours, respectively) and weekend (5.4 hours and 5.1 hours, respectively) [13].

Although physical inactivity and SB are distinct constructs [14], PA, including light-intensity (i.e., 1.6 to 2.9 METs), moderate-intensity (i.e., 3 to 5.9 METs), and vigorous-intensity (≥6 METs) activities, and SB exist on the same energy expenditure spectrum [15]. In fact, if we assume adults sleep an average of eight hours each night, only around 2% of our waking time is required to meet PA guidelines for aerobic activity in the UK [3]. Meanwhile, the remaining 98% of our waking time is spent in SB and light-intensity activity, including static (e.g., standing) and ambulatory activities [14]. As a result, it is important to explore activities across the energy expenditure spectrum, not merely the least practiced moderate-to-vigorous-intensity physical activity (MVPA). Furthermore, PA participation modifies the effect of SB on mortality, where higher amounts of MVPA can mitigate all-cause, cardiovascular disease, and cancer related deaths [2,10,16]. For example, high levels of moderate-intensity activity (i.e., 60 to 75 minutes per day) has been found to eliminate the risk of mortality associated with high sitting time, whilst only attenuating the risk associated with high television-viewing time [16].

In the UK, physical inactivity accounts for one in six deaths and has an estimated annual cost of £7.4 billion, including £0.9 billion to the National Health Service (NHS) alone [17]. Moreover, prolonged SB (≥6 hours each day) was estimated to cost the NHS £0.8 billion between 2016 and 2017, which, if eliminated, could have avoided 69,276 deaths [18]. Considering the prevalence and economic and social burden of physical inactivity and SB, it is important to identify populations that are less physically active and/or more sedentary and characterise and understand these contexts to develop targeted interventions which promote PA.

Throughout the life course, there are major life events and transitions that are associated with reduced PA, which may have further implications for health and wellbeing [19]. Life events 'mark the beginning or end of a specific status. A status is a nominal variable with at least two levels' [20]. For

For peer review only - http://bmjopen.bmj.com/site/about/guidelines.xhtml

example, the life event of parenthood, or having a child, could be conceptualised to involve multiple consecutive shifts in status from preconception (or non-parent) to pregnant, postpartum (until six months after giving birth [21]), then finally into parenthood (or parent). Notably, life events can bring about periods of biological, psychological, and/or social adaption and readjustment which can subsequently lead to behaviour change, such as reduced PA participation [22]. Life transitions are conceptualised through these adaptive periods and reflect the processual character and temporality of life events, including their anticipation (to the extent that they can be expected), history, and eventual aftermath [22]. As a result, both life events and transitions are inherently interconnected yet differ in their temporal characteristics. For instance, life events are singular occurrences which mark a change in status, whilst life transitions represent the duration of that given status change [20,22]. These conceptualisations of life events and transitions exclude minor life events, such as daily hassles; slow transitions without clearly identifiable life events, like ageing; and non-events, such as not having a child [20,22].

Parenthood, or having a child, is one such life event and transition that has been identified as a critical transformative experience and window for adult health and wellbeing and intervention [23]. Moreover, having a child is often interconnected with other life events and transitions, including changes to relationship status, and living situation, which may further compound these effects [24]. As a result, the impact of having a child on PA should be characterised and understood in its entirety, from the preconception period, throughout pregnancy, the postpartum period, and beyond into parenthood, and contextually among other related life events and transitions.

The latest reviews of existing evidence exploring the impact of having a child on PA by Bellows-Riecken and Rhodes [25] and Corder et al. [26] suggest that the transition into parenthood, becoming a parent, generally reduces PA participation, particularly in new mothers. Although both reviews provide valuable characterisations and understandings of PA during this time, their scope focusses on the differences between parents and non-parents and does not consider the other relevant changes in status embedded in the 'having' of a child, such as becoming pregnant and entering the postpartum period, with their inherent temporal and processual characteristics. In addition, although both reviews include quantitative and qualitative evidence, these were only sourced from published literature, consequently missing the potentially rich insights afforded by grey literature. Both reviews also chose to forgo the characterisation and understanding of the relationship between PA and SB. What's more, these reviews included international sources of evidence from a variety of countries, namely the United States, Australia, Canada, and the UK, among others from Europe. Whilst these findings may be generalisable to western societies en masse, it is important to ensure findings are culturally and contextually relevant to the UK, making them more applicable and practicable within this setting. Given that there were 585,195 live births in England in 2020 [27], it is important to provide an overview of available evidence exploring the impact of having a child in the UK.

Recent evidence exploring the impact of having a child on PA by intensity, including SB, indicates that parents participate in significantly more light-intensity activity but less MVPA and SB, compared to those without children [28-29]. For example, in the study by Gaston et al. [28], mothers took part in more light-intensity activity ($p = 0.001$) but less MVPA ($p = 0.000$) than women without children. Meanwhile, fathers only engaged in more light-intensity activity, compared to men without children ($p = 0.000$). These associations differed by the number and age of children. For instance, mothers with one child, new mothers, participated in more light-intensity activity ($p = 0.005$) than women without children, while mothers with two or three or more children took part in less MVPA ($p = 0.00$ and $p = 0.005$, respectively). Notably, mothers whose youngest child was aged 12 to 15 engaged in more SB ($p = 0.017$), compared to women without children, whilst mothers whose youngest child was aged less than six participated in more light-intensity activity ($p = 0.000$) and less MVPA ($p = 0.000$). In fathers, those with two children took part in more light-intensity activity than men without children ($p = 0.000$). Furthermore, fathers whose youngest child was aged less than six engaged in more light-intensity activity ($p = 0.000$) but less MVPA ($p = 0.000$), compared to men without children. Considering

For peer review only - http://bmjopen.bmj.com/site/about/guidelines.xhtml

that parents participate in more light-intensity activity and less MVPA and SB than those without children, it is necessary to review available evidence exploring PA by intensity and examine the relationship between PA and SB across the entirety of having a child, from the preconception period, throughout pregnancy, the postpartum period, and into parenthood.

Preliminary searches broadened our knowledge of available evidence and informed the formulation of research questions. We conducted searches of Medline and Google Scholar to identify reviews exploring the impact of having a child on PA in the UK. The search terms were 'having a child' AND 'physical activity' AND 'review' AND ('United Kingdom' OR 'UK'). No relevant reviews were identified. We conducted further searches to identify relevant primary research studies by removing the search term relating to reviews. No studies were identified from Medline. Only one study was identified as relevant from the first 100 of 3,080 results from Google Scholar [30]. This study by Werneck et al. [30] examined the association between family-related life-events (cohabitation/marriage and becoming a parent) and change in PA in the UK. Findings from this study suggest that becoming a parent was associated with a decline in physical activity among men ($\beta = -0.234$, 95%CI -0.396 to -0.072), but not women ($\beta = 0.126$, 95% CI -0.048 to 0.301). Specifically, men who became fathers while cohabiting ($\beta = -0.201$, 95% CI -0.383 to -0.020) and without cohabiting ($\beta = -0.937$, 95% CI -1.623 to -0.250) experienced greater declines in PA compared to those remaining single and without children, where non-cohabiting fathers experienced the greatest decline. These associations did not differ by the age of children. Since no relevant reviews were identified and a dearth of primary research studies, we aim to provide an overview of available evidence exploring the impact of having a child on PA in the UK.

METHODS AND ANALYSIS

We will conduct a review of available evidence exploring the impact of having a child on PA in the UK using best-practice scoping review methodologies. Although there is no consensus definition for scoping reviews [31-32], it is a form of literature review which can map key concepts and available evidence within a given topic area, exploring the extent, range, and nature of existing evidence [33-34]. Moreover, scoping reviews can define and clarify existing concepts, summarise and disseminate findings to key stakeholders, such as practitioners and policymakers, identify knowledge gaps, and determine the main priorities for future research [33-34].

A scoping review, rather than a systematic review, is more congruent with the aims and purposes of this study. Specifically, scoping reviews use broad research questions to provide an overview of the available evidence [33]. Meanwhile, systematic reviews use focussed research questions to identify the best evidence. Despite being useful as a precursor to systematic reviews, scoping reviews are legitimate in their own right as standalone studies and are not required to be followed up with a systematic review [33]. Furthermore, although scoping reviews are not, by name, systematic, they do follow a rigorous systematic process [35-36]. Unlike systematic reviews, it is important to recognise that best-practice methodological frameworks for scoping reviews do not recommend critical appraisal of individual sources of evidence, although this is still possible with reasonable justification [35-38]. Plainly, scoping reviews should not be considered a rapid and cheap alternative to systematic reviews, where they map the breadth of available literature and, as such, require a substantial amount of time and resources, including labour and finances [33].

We will use best-practice scoping review methodological frameworks by Arksey and O'Malley [33], adapted by Levac et al. [34] and the Joanna Briggs Institute (JBI) [36-37], to guide the conduct of this review. This is an iterative process by which research questions and methods may be further developed with the emergent evidence. We will report any deviation from this protocol in the final review. In addition, we will use the Preferred Reporting Items for Systematic reviews and Meta-Analysis extension for Scoping Reviews (PRISMA-ScR) to guide the reporting of this protocol as detailed in **supplementary material 1** [39]. Furthermore, methods were informed by previous scoping

For peer review only - http://bmjopen.bmj.com/site/about/guidelines.xhtml

reviews in PA for health research [22,40-42]. This scoping review is registered with the Open Science Framework (OSF) to enhance the rigorousness and transparency of this research process.

Patient and public involvement

Patients and members of the public were not involved in the preparation of this protocol. However, key stakeholders, including practitioners and policymakers, with their unique expertise and perspectives, will be consulted to give comment on findings to inform the main priorities for future research.

Stage 1: Identifying the research questions

The following broad research questions were informed by preliminary searches and interdisciplinary discussions within the research team. Moreover, research questions were formulated using the P.C.C. (Population, Concept, Context) mnemonic which will help to identify relevant studies and establish appropriate eligibility criteria [37].

- What is known about the impact of having a child on PA in the UK?
    - What is known about the relationship between PA and SB in this field?
- What are the knowledge gaps in this field?
- What are the main priorities for future research in this field?

Stage 2: Identifying relevant studies

Eligibility criteria

The following a priori eligibility criteria was informed by preliminary searches, interdisciplinary discussions within the research team, and research questions.

- Participants have to be adults that are reported to have been planning to have (i.e., preconception, non-parents), having (i.e., pregnant), have had (i.e., postpartum and into parenthood, parents), or planned to have (i.e., stillbirth) a child.
- Participants can be from any group, including general and specific populations (e.g., those recruited based on their living with a physical or mental illness or condition).
- Participants can be of any sex, ethnicity, parity, relationship status, and socio-economic position [43].
- Findings must explore the impact of having a child on PA, but not the impact of PA on health and wellbeing.
- Findings must report data on PA and/or sedentary behaviour which was assessed either quantitatively (i.e., device-derived or through self-report) and/or qualitatively (e.g., attitudes, views, perspectives, experiences, etc.).
- Studies can be published literature (e.g., primary research studies, including reviews) or grey literature (e.g., unpublished dissertations/theses or conference proceedings).
- Studies must be of quantitative observational (e.g., cross-sectional, prospective cohort), qualitative, or mixed methods study design.
- Studies must not have an experimental study design (e.g., randomised controlled trials [RCTs]) evaluating adherence and/or acceptability of interventions, or secondary analysis thereof; guidelines, opinion pieces (e.g., commentaries or editorials); or popular press articles (e.g., magazine or newspaper articles).
- Studies must be conducted with data generated exclusively within the UK, including England, Northern Ireland, Scotland, and Wales.
- Studies must be written in the English language.

Search strategy

Step 1: An initial limited search

We conducted an initial limited search of Medline (Ovid) and Google Scholar (Advanced Search) for relevant reviews. Given that there were no reviews exploring the impact of having a child on PA in the

For peer review only - http://bmjopen.bmj.com/site/about/guidelines.xhtml

UK, we expanded search terms to allow for those using data generated elsewhere, including 'having a child' AND 'physical activity' AND 'review'. The two records identified from Medline and the first 100 of the 6,720 records identified from Google Scholar were reviewed. A total of five reviews were identified as relevant [22,25,44-46]. We also conducted a search of ProQuest to identify any similar dissertations but did not retrieve any records.

Step 2: Identifying keywords and search terms
We used keywords and index terms featured within the titles and abstracts of the relevant reviews to inform main searches. Main searches will identify studies exploring the impact of having a child on PA in the UK, from the preconception period, throughout pregnancy, the postpartum period, and into parenthood, from inception to February 2022. We first prepared an a priori search strategy for published literature within the Medline electronic bibliographic database, with Boolean operators and Medical Subject Headings (i.e., MeSH terms). We then adapted this search strategy for searches within CINAHL (EBSCOhost), Embase (Ovid), PsycINFO (Ovid), and Web of Science databases (Clarivate). All search strategies are detailed in **supplementary material 2**. Search terms will relate to those status shifts involved in 'having a child' in an attempt to remain inclusive, where having a child can impact a variety of populations, including those of differing sex, ethnicity, parity, relationship status, and socio-economic position [43]. Moreover, search terms will relate to the main concept of 'physical activity', including 'sedentary behaviour', and the context of the 'United Kingdom'. We have made a pragmatic decision to only search for studies which have these search terms in the title and/or abstract due to the practical constraints of this review.

We will use a similar search strategy for grey literature, where the search terms used for Medline has been adapted for Google Scholar and ProQuest (**supplementary material 2**). In Google Scholar, this will involve a reduced set of search terms and multiple searches due to the limitation on characters. Moreover, in Google Scholar, it is only possible to search for studies which have these search terms in the title. We will also search websites of organisations (e.g., Best Beginning's, Tommy's) involved in the commissioning, undertaking, or cataloguing of research in this field and, if appropriate, we will make contact by email.

Step 3: Further searching of references and citations
We will conduct backwards citation tracking of included studies to identify further potentially eligible studies. Considering that scoping reviews are typically iterative, additional search terms and information sources (e.g., electronic bibliographic databases and websites) may be added to existing search strategies at any time throughout the research process. We may also contact corresponding authors of included studies to request further information. We will make search strategies available from the OSF registration. We will import references of identified studies into Endnote X9.2 reference management software and organise them by database of origin, with duplicates removed.

Stage 3: Study selection

Titles and abstracts of identified studies will be predominantly screened for eligibility by one researcher (MN). A second researcher (JB) will independently complete this same process on a random sample of 10% of titles and abstracts. If the same screening decisions are taken by both researchers for 95% of studies, then MN will screen the remaining titles and abstracts. Meanwhile, if the same decisions are taken for less than 95% of studies, then MN and JB will meet to discuss these decisions with reference to the eligibility criteria and repeat this process. Any conflicts after this point will be resolved by a third researcher (CF/RP). If there is no consensus between these three parties, then we will include the study in the review. All researchers will meet to discuss the eligibility criteria before embarking on this process to minimise these occurrences.

For peer review only - http://bmjopen.bmj.com/site/about/guidelines.xhtml

Stage 4: Charting the data

Data charting of included studies will be predominantly conducted by MN. A data charting form will be employed, using the a priori headings detailed below [37]. Given that scoping reviews are typically iterative, additional headings may be added to the form throughout the research process. The data charting process will involve MN charting 90% of studies and JB charting 10% of studies, with JB checking 10% of MN's data and vice versa. If there are considerable differences between researchers, MN and JB will meet to discuss decisions with reference to the data charting form and rectify any issues. Any conflicts will be resolved by CF/RP during group meetings. If a full text article cannot be retrieved, MN will attempt to gain access through the university library and by contacting corresponding authors. If the full text article is not accessible, the study will be excluded.

- Author(s).
- Year of publication.
- Source and country of origin
- Aims/purpose.
- Study population and sample size (if applicable).
- Methodology/methods
- Concept
- Findings and details of these (e.g., how measured) (if applicable).
- Key findings by theme that relate to research questions.

Stage 5: Collating, summarising, and reporting the findings

We will conduct a numeric analysis of included studies to describe review decisions, findings from searches, removal of duplicates, study selection, full text retrieval, and backwards citation tracking [37]. We will present these findings using a study flow diagram. In addition, we will map data in a tabular and diagrammatic format by period of publication, country of origin, study type, and themes. Considering that scoping reviews are typically an iterative process, we may include additional categories of data. We will also conduct a thematic analysis of included studies using NVivo 12 qualitative data analysis software to provide a description of key concepts relevant to research questions and findings by theme. We will analyse these findings whilst being open to socio-demographic differences in the target population, such as sex, ethnicity, parity, relationship status, and socio-economic position [43]. Furthermore, we will use the PRISMA-ScR framework to guide the reporting of this study [39].

Critical appraisal of individual sources of evidence

We will not conduct critical appraisal of individual sources of evidence, where this is consistent with methodological frameworks for scoping reviews [33].

ETHICS AND DISSEMINATION

Ethical approval will not be required for this scoping review. This review will provide a novel insight into the impact of having a child on PA in the UK. Specifically, we will map key concepts and available evidence, identify knowledge gaps, and determine the main priorities for future research. We will subsequently summarise and submit findings for peer review and publication in a relevant open-access journal. Moreover, we will develop an infographic to be disseminated at conferences and online via professional social media channels, such as Twitter. Importantly, findings from this study will be reported regardless of the nature of the relationships between having a child and PA in the UK. As previously stated, we also aim to consult with key stakeholders, including practitioners and policymakers, to comment on findings and provide greater insight with their unique expertise and perspectives [34,47].

For peer review only - http://bmjopen.bmj.com/site/about/guidelines.xhtml

ACKNOWLEDGEMENTS
The authors would like to thank Dr Joey Murphy for his guidance in identifying relevant literature pertaining to best-practice methodological frameworks for scoping reviews. We would also like to thank Jack Brazier (JB), a non-author, for accepting the role of second researcher within the review selection and data charting processes.

CONTRIBUTORS
MN, CF, RP, and FS formulated the research questions for this scoping review. CF identified the methodological frameworks and methods. MN prepared the methods. MN, CF, and RP contributed to the development of the search strategy. MN wrote the first and subsequent drafts of the manuscript. All authors reviewed and approved the manuscript.

COMPETING INTERESTS
None declared.

FUNDING
This study was part of MN's PhD research at the Centre for Exercise, Nutrition, and Health Sciences, School for Policy Studies, University of Bristol. MN is a PhD Researcher with the South West Doctoral Training Partnership (SWDTP) which is funded by the Economic and Social Research Council (ESRC) (grant number: ES/P000630/1).

REFERENCES
1 Capsersen CJ, Powell KE, Christenson GM. Physical activity, exercise, and physical fitness: definitions and distinctions for health-related research. *Public Health Rep* 1985 Mar-Apr;100(2):126–30.

2 2018 Physical Activity Guidelines Advisory Committee. Physical Activity Guidelines Advisory Committee Scientific Report. Washington DC: United States Department of Health and Human Services. 2018.

3 Department of Health and Social Care. UK Chief Medical Officers' Physical Activity Guidelines. 2019.

[dataset] [4] NHS Digital. Data from: Health Survey for England, 2016: Adult physical activity – tables. Health Survey for England, 2016, December 13, 2017.

5 Department of Health. Health Survey (NI) First Results 2016/17. 2017.

[dataset] [6] Scottish Government. Data from: Chapter 5 Tables. Scottish Health Survey – telephone survey – August/September 2020: main report, January 26, 2021.

[dataset] [7] Welsh Government. Data from: Adult lifestyles by local authority and health board, 2020-21 onwards. StatsWales, July 2022.

[dataset] [8] Scottish Government. Data from: Chapter 7 – Physical Activity Tables. Scottish Health Survey 2019 – volume 1: main report, September 29, 2020.

9 Tremblay MS, Aubert S, Barnes JD, Saunders TJ, Carson V, Latimer-Cheung AE, Chastin SF, Altenburg TM, Chinapaw MJ. Sedentary behavior research network (SBRN)–terminology consensus project process and outcome. *Int J Behav Nutr Phys Act* 2017 Jun 10;14(1):75. doi: 10.1186/s12966-017-0525-8

10 Ekelund U, Brown WJ, Steene-Johannessen J, Fagerland MW, Owen N, Powell KE, Bauman AE, Lee IM. Do the associations of sedentary behaviour with cardiovascular disease mortality and cancer mortality differ by physical activity level? A systematic review and harmonised meta-analysis of data from 850 060 participants. *Br J Sports Med* 2019 Jul;53(14):886-894. doi: 10.1136/bjsports-2017-098963

For peer review only - http://bmjopen.bmj.com/site/about/guidelines.xhtml

11 Ekelund U, Tarp J, Steene-Johannessen J, Hansen BH, Jefferis B, Fagerland MW, Whincup P, Diaz KM, Hooker SP, Chernofsky A, Larson MG. Dose-response associations between accelerometry measured physical activity and sedentary time and all cause mortality: systematic review and harmonised meta-analysis. *BMJ* 2019 Aug 21;366:l4570. doi: 10.1136/bmj.l4570

12 Patterson R, McNamara E, Tainio M, de Sá TH, Smith AD, Sharp SJ, Edwards P, Woodcock J, Brage S, Wijndaele K. Sedentary behaviour and risk of all-cause, cardiovascular and cancer mortality, and incident type 2 diabetes: a systematic review and dose response meta-analysis. *Eur J Epidemiol* 2018 Sep;33(9):811-829. doi: 10.1007/s10654-018-0380-1

13 Scholes S, Mindell J. Health Survey for England – 2012: Chapter 2, Physical activity in adults. 2013

14 van der Ploeg HP, Hillsdon M. Is sedentary behaviour just physical inactivity by another name?. Int *J Behav Nutr Phys Act* 2017 Oct 23;14(1):142. doi: 10.1186/s12966-017-0601-0

15 Ainsworth BE, Haskell WL, Herrmann SD, Meckes N, Bassett DR, Tudor-Locke C, Greer JL, Vezina J, Whitt-Glover MC, Leon AS. 2011 Compendium of Physical Activities: a second update of codes and MET values. *Med Sci Sports Exerc* 2011 Aug;43(8):1575-81. doi: 10.1249/MSS.0b013e31821ece12

16 Ekelund U, Steene-Johannessen J, Brown WJ, Fagerland MW, Owen N, Powell KE, Bauman A, Lee IM, Lancet Physical Activity Series 2 Executive Committee, Lancet Sedentary Behaviour Working Group. Does physical activity attenuate, or even eliminate, the detrimental association of sitting time with mortality? A harmonised meta-analysis of data from more than 1 million men and women. *Lancet*. 2016 Sep 24;388(10051):1302-10. doi: 10.1016/S0140-6736(16)30370-1

17 Office for Health Improvement & Disparities. Physical activity: applying All Our Health. 2022.

18 Heron L, O'Neill C, McAneney H, Kee F, Tully MA. Direct healthcare costs of sedentary behaviour in the UK. *J Epidemiol Community Health* 2019 Jul;73(7):625-629. doi: 10.1136/jech-2018-211758

19 Allender S, Hutchinson L, Foster C. Life-change events and participation in physical activity: a systematic review. *Health Promot Int* 2008 Jun;23(2):160-72. doi: 10.1093/heapro/dan012

20 Luhmann M, Hofmann W, Eid M, Lucas RE. Subjective well-being and adaptation to life events: a meta-analysis. *J Pers Soc Psychol* 2012 Mar;102(3):592-615. doi: 10.1037/a0025948

21 Chauhan G, Tadi P. Physiology, Postpartum Changes. Available: https://www.ncbi.nlm.nih.gov/books/NBK555904/ [Accessed 29 Jul 2022].

22 Gropper H, John JM, Sudeck G, Thiel A. The impact of life events and transitions on physical activity: A scoping review. *PLoS One* 2020 Jun 22;15(6):e0234794. doi: 10.1371/journal.pone.0234794

23 Saxbe D, Rossin-Slater M, Goldenberg D. The transition to parenthood as a critical window for adult health. *Am Psychol* 2018 Dec;73(9):1190-1200. doi: 10.1037/amp0000376

24 Hull EE, Rofey DL, Robertson RJ, Nagle EF, Otto AD, Aaron DJ. Influence of marriage and parenthood on physical activity: a 2-year prospective analysis. *J Phys Act Health* 2010 Sep;7(5):577-83. doi: 10.1123/jpah.7.5.577

25 Bellows-Riecken KH, Rhodes RE. A birth of inactivity? A review of physical activity and parenthood. *Prev Med* 2008 Feb;46(2):99-110. doi: 10.1016/j.ypmed.2007.08.003

26 Corder K, Winpenny EM, Foubister C, Guagliano JM, Hartwig XM, Love R, Clifford Astbury C, van Sluijs EM. Becoming a parent: A systematic review and meta-analysis of changes in BMI, diet, and physical activity. *Obes Rev* 2020 Apr;21(4):e12959. doi: 10.1111/obr.12959

[dataset] [27] Office for National Statistics. Data from: Births in England and Wales: summary tables. October 14, 2021.

For peer review only - http://bmjopen.bmj.com/site/about/guidelines.xhtml

28 Gaston A, Edwards SA, Doelman A, Tober JA. The impact of parenthood on Canadians' objectively measured physical activity: an examination of cross-sectional population-based data. *BMC Public Health.* 2014 Nov 3;14:1127. doi: 10.1186/1471-2458-14-1127

29 Rhodes RE, Blanchard CM, Benoit C, Levy-Milne R, Naylor PJ, Symons Downs D, Warburton DE. Physical activity and sedentary behavior across 12 months in cohort samples of couples without children, expecting their first child, and expecting their second child. *J Behav Med* 2014 Jun;37(3):533-42. doi: 10.1007/s10865-013-9508-7

30 Werneck AO, Winpenny EM, Van Sluijs EM, Corder K. Cohabiting and becoming a parent: associations with changes in physical activity in the 1970 British cohort study. *BMC Public Health* 2020 Jul 10;20(1):1085. doi: 10.1186/s12889-020-09187-2

31 Colquhoun HL, Levac D, O'Brien KK, Straus S, Tricco AC, Perrier L, Kastner M, Moher D. Scoping reviews: time for clarity in definition, methods, and reporting. *J Clin Epidemiol* 2014 Dec;67(12):1291-4. doi: 10.1016/j.jclinepi.2014.03.013

32 Peterson J, Pearce PF, Ferguson LA, Langford CA. Understanding scoping reviews: Definition, purpose, and process. *J Am Assoc Nurse Pract* 2017 Jan;29(1):12-6. doi: 10.1002/2327-6924.12380

33 Arksey H, O'Malley L. Scoping studies: towards a methodological framework. *Int J Soc Res Methodol*. 2005 Feb;8(1):19-32. doi: 10.1080/1364557032000119616

34 Levac D, Colquhoun H, O'Brien KK. Scoping studies: advancing the methodology. *Implement Sci*. 2010 Sep 20;5:69. doi: 10.1186/1748-5908-5-69

35 Polit DF, Beck CT. Systematic reviews of research evidence: Meta-analysis, metasynthesis, and mixed studies review. In: Polit DF, Beck CT, 9th ed. Nursing research: Generating and assessing evidence for nursing practice. Philadeplhia, PA. 2012.

36 Peters MD, Marnie C, Tricco AC, Pollock D, Munn Z, Alexander L, McInerney P, Godfrey CM, Khalil H. Updated methodological guidance for the conduct of scoping reviews. *JBI Evid Synth* 2020 Oct;18(10):2119-2126. doi: 10.11124/JBIES-20-00167

37 Peters MD, Godfrey CM, Khalil H, McInerney P, Parker D, Soares CB. Guidance for conducting systematic scoping reviews. *Int J Evid Based Healthc* 2015 Sep;13(3):141-6. doi: 10.1097/XEB.0000000000000050

38 Khalil H, Peters M, Godfrey CM, McInerney P, Soares CB, Parker D. An evidence-based approach to scoping reviews. *Worldviews Evid Based Nurs*. 2016 Apr;13(2):118-23. doi: 10.1111/wvn.12144

39 Tricco AC, Lillie E, Zarin W, O'Brien KK, Colquhoun H, Levac D, Moher D, Peters MD, Horsley T, Weeks L, Hempel S. PRISMA extension for scoping reviews (PRISMA-ScR): checklist and explanation. *Ann Intern Med* 2018 Oct 2;169(7):467-473. doi: 10.7326/M18-0850

40 Murray AD, Daines L, Archibald D, Hawkes RA, Schiphorst C, Kelly P, Grant L, Mutrie N. The relationships between golf and health: a scoping review. *Br J Sports Med* 2017 Jan;51(1):12-19. doi: 10.1136/bjsports-2016-096625

41 Griffin SA, Perera NK, Murray A, Hartley C, Fawkner SG, Kemp SP, Stokes KA, Kelly P. The relationships between rugby union, and health and well-being: a scoping review. *Br J Sports Med* 2021 Mar;55(6):319-326. doi: 10.1136/bjsports-2020-102085

42 Williamson C, Baker G, Mutrie N, Niven A, Kelly P. Get the message? A scoping review of physical activity messaging. *Int J Behav Nutr Phys Act* 2020 Apr 15;17(1):51. doi: 10.1186/s12966-020-00954-3

43 Galobardes B, Lynch J, Smith GD. Measuring socioeconomic position in health research. *Br Med Bull* 2007;81-82:21-37. doi: 10.1093/bmb/ldm001

For peer review only

44 Engberg E, Alen M, Kukkonen-Harjula K, Peltonen JE, Tikkanen HO, Pekkarinen H. Life events and change in leisure time physical activity. *Sports Med* 2012 May 1;42(5):433-47. doi: 10.2165/11597610-000000000-00000

45 Condello G, Puggina A, Aleksovska K, Buck C, Burns C, Cardon G, Carlin A, Simon C, Ciarapica D, Coppinger T, Cortis C. Behavioral determinants of physical activity across the life course: a "DEterminants of DIet and Physical ACtivity"(DEDIPAC) umbrella systematic literature review. *Int J Behav Nutr Phys Act* 2017 May 2;14(1):58. doi: 10.1186/s12966-017-0510-2

46 Stults-Kolehmainen MA, Sinha R. The effects of stress on physical activity and exercise. *Sports Med* 2014 Jan;44(1):81-121. doi: 10.1007/s40279-013-0090-5

47 Pham MT, Rajić A, Greig JD, Sargeant JM, Papadopoulos A, McEwen SA. A scoping review of scoping reviews: advancing the approach and enhancing the consistency. *Res Synth Methods* 2014 Dec;5(4):371-85. doi: 10.1002/jrsm.1123

For peer review only

For peer review only - http://bmjopen.bmj.com/site/about/guidelines.xhtml

Preferred Reporting Items for Systematic reviews and Meta-Analyses extension for Scoping Reviews (PRISMA-ScR) Checklist

SECTION	ITEM	PRISMA-ScR CHECKLIST ITEM	REPORTED ON PAGE #
**TITLE**			
Title	1	Identify the report as a scoping review.	Title page
**ABSTRACT**			
Structured summary	2	Provide a structured summary that includes (as applicable): background, objectives, eligibility criteria, sources of evidence, charting methods, results, and conclusions that relate to the review questions and objectives.	1
**INTRODUCTION**			
Rationale	3	Describe the rationale for the review in the context of what is already known. Explain why the review questions/objectives lend themselves to a scoping review approach.	2-4
Objectives	4	Provide an explicit statement of the questions and objectives being addressed with reference to their key elements (e.g., population or participants, concepts, and context) or other relevant key elements used to conceptualize the review questions and/or objectives.	4-5
**METHODS**			
Protocol and registration	5	Indicate whether a review protocol exists; state if and where it can be accessed (e.g., a Web address); and if available, provide registration information, including the registration number.	1
Eligibility criteria	6	Specify characteristics of the sources of evidence used as eligibility criteria (e.g., years considered, language, and publication status), and provide a rationale.	5
Information sources*	7	Describe all information sources in the search (e.g., databases with dates of coverage and contact with authors to identify additional sources), as well as the date the most recent search was executed.	5-6
Search	8	Present the full electronic search strategy for at least 1 database, including any limits used, such that it could be repeated.	6, supplementary material 2
Selection of sources of evidence†	9	State the process for selecting sources of evidence (i.e., screening and eligibility) included in the scoping review.	6
Data charting process‡	10	Describe the methods of charting data from the included sources of evidence (e.g., calibrated forms or forms that have been tested by the team before their use, and whether data charting was done independently or in duplicate) and any processes for obtaining and confirming data from investigators.	7
Data items	11	List and define all variables for which data were sought and any assumptions and simplifications made.	5, 7
Critical appraisal of individual	12	If done, provide a rationale for conducting a critical appraisal of included sources of evidence; describe	7

SECTION	ITEM	PRISMA-ScR CHECKLIST ITEM	REPORTED ON PAGE #
sources of evidence§		the methods used and how this information was used in any data synthesis (if appropriate).	
Synthesis of results	13	Describe the methods of handling and summarizing the data that were charted.	7
**RESULTS**			
Selection of sources of evidence	14	Give numbers of sources of evidence screened, assessed for eligibility, and included in the review, with reasons for exclusions at each stage, ideally using a flow diagram.	NA
Characteristics of sources of evidence	15	For each source of evidence, present characteristics for which data were charted and provide the citations.	NA
Critical appraisal within sources of evidence	16	If done, present data on critical appraisal of included sources of evidence (see item 12).	NA
Results of individual sources of evidence	17	For each included source of evidence, present the relevant data that were charted that relate to the review questions and objectives.	NA
Synthesis of results	18	Summarize and/or present the charting results as they relate to the review questions and objectives.	NA
**DISCUSSION**			
Summary of evidence	19	Summarize the main results (including an overview of concepts, themes, and types of evidence available), link to the review questions and objectives, and consider the relevance to key groups.	NA
Limitations	20	Discuss the limitations of the scoping review process.	1,4-5
Conclusions	21	Provide a general interpretation of the results with respect to the review questions and objectives, as well as potential implications and/or next steps.	NA
**FUNDING**			
Funding	22	Describe sources of funding for the included sources of evidence, as well as sources of funding for the scoping review. Describe the role of the funders of the scoping review.	8

JBI = Joanna Briggs Institute; PRISMA-ScR = Preferred Reporting Items for Systematic reviews and Meta-Analyses extension for Scoping Reviews.

* Where *sources of evidence* (see second footnote) are compiled from, such as bibliographic databases, social media platforms, and Web sites.

† A more inclusive/heterogeneous term used to account for the different types of evidence or data sources (e.g., quantitative and/or qualitative research, expert opinion, and policy documents) that may be eligible in a scoping review as opposed to only studies. This is not to be confused with *information sources* (see first footnote).

‡ The frameworks by Arksey and O'Malley (6) and Levac and colleagues (7) and the JBI guidance (4, 5) refer to the process of data extraction in a scoping review as data charting.

§ The process of systematically examining research evidence to assess its validity, results, and relevance before using it to inform a decision. This term is used for items 12 and 19 instead of "risk of bias" (which is more applicable to systematic reviews of interventions) to include and acknowledge the various sources of evidence that may be used in a scoping review (e.g., quantitative and/or qualitative research, expert opinion, and policy document).

From: Tricco AC, Lillie E, Zarin W, O'Brien KK, Colquhoun H, Levac D, et al. PRISMA Extension for Scoping Reviews (PRISMAScR): Checklist and Explanation. Ann Intern Med. 2018;169:467–473. doi: 10.7326/M18-0850.

For peer review only - http://bmjopen.bmj.com/site/about/guidelines.xhtml

SUPPLEMENTARY MATERIAL 2: SEARCH STRATEGIES

PUBLISHED LITERATURE SEARCH STRATEGIES

CINAHL (EBSCOhost)

In title:

(“Physical activity” OR Exercise OR “Sedentary behaviour”) AND (“Having a child” OR “Having children” OR Childbearing OR Preconception OR Pregnancy OR Postpartum OR Post-partum OR Parenthood OR Motherhood OR Fatherhood) AND (England OR “Northern Ireland” OR Scotland OR Wales OR “United Kingdom” OR UK)

In abstract:

(“Physical activity” OR Exercise OR “Sedentary behaviour”) AND (“Having a child” OR “Having children” OR Childbearing OR Preconception OR Pregnancy OR Postpartum OR Post-partum OR Parenthood OR Motherhood OR Fatherhood) AND (England OR “Northern Ireland” OR Scotland OR Wales OR “United Kingdom” OR UK)

Embase (Embase 1974 to 2022 February 04)

1. Physical activity.ti,ab.
2. Physical activity/
3. Exercise.ti,ab.
4. Exercise/
5. Sedentary behaviour.ti,ab.
6. Having a child.ti,ab.
7. Having children.ti,ab.
8. Childbearing.ti,ab.
9. Preconception.ti,ab.
10. Pregnancy.ti,ab.
11. Pregnancy/
12. Postpartum.ti,ab.
13. Post-partum.ti,ab.
14. Parenthood.ti,ab.
15. Parenthood/
16. Motherhood.ti,ab.
17. Fatherhood.ti,ab.
18. England.ti,ab.
19. England/
20. Northern Ireland.ti,ab.
21. Northern Ireland/
22. Scotland.ti,ab.
23. Scotland/
24. Wales.ti,ab.
25. Wales/
26. United Kingdom.ti,ab.
27. United Kingdom/
28. UK.ti,ab.
29. 1 OR 2 OR 4 OR 4 OR 5
30. 6 OR 7 OR 8 OR 9 OR 10 OR 11 OR 12 OR 13 OR 14 OR 15 OR 16 OR 17
31. 18 OR 19 OR 20 OR 21 OR 22 OR 23 OR 24 OR 25 OR 26 OR 27 OR 28
32. 29 AND 30 AND 31

For peer review only

Medline (Ovid MEDLINE® 1946 to present)

1. Physical activity.ti,ab.
2. Exercise.ti,ab.
3. Exercise/
4. Sedentary behaviour.ti,ab.
5. Sedentary behaviour/
6. Having a child.ti,ab.
7. Having children.ti,ab.
8. Childbearing.ti,ab.
9. Preconception.ti,ab.
10. Pregnancy.ti,ab.
11. Pregnancy/
12. Postpartum.ti,ab.
13. Post-partum.ti,ab.
14. Parenthood.ti,ab.
15. Motherhood.ti,ab.
16. Fatherhood.ti,ab.
17. England.ti,ab.
18. England/
19. Northern Ireland.ti,ab.
20. Northern Ireland/
21. Scotland.ti,ab.
22. Scotland/
23. Wales.ti,ab.
24. Wales/
25. United Kingdom.ti,ab.
26. United Kingdom/
27. UK.ti,ab.
28. 1 OR 2 OR 3 OR O4 OR 5
29. 6 OR 7 OR 8 OR 9 OR 10 OR 11 OR 12 OR 13 OR 14 OR 15 OR 16
30. 17 OR 18 OR 19 OR 20 OR 21 OR 22 OR 23 OR 24 OR 25 OR 26 OR 27
31. 28 AND 29 AND 30

PsycInfo (APA PsychInfo 1806 to January Week 5 2022)

1. Physical activity.ti,ab.
2. Physical activity/
3. Exercise.ti,ab.
4. Exercise/
5. Sedentary behaviour.ti,ab.
6. Sedentary behaviour/
7. Having a child.ti,ab.
8. Having children.ti,ab.
9. Childbearing.ti,ab.
10. Preconception.ti,ab.
11. Pregnancy.ti,ab.
12. Pregnancy/
13. Postpartum.ti,ab.
14. Post-partum.ti,ab.
15. Parenthood.ti,ab.
16. Motherhood.ti,ab.
17. Fatherhood.ti,ab.
18. England.ti,ab.

For peer review only

19. Northern Ireland.ti,ab.
20. Scotland.ti,ab.
21. Wales.ti,ab.
22. United Kingdom.ti,ab.
23. UK.ti,ab.
24. 1 or 2 or 3 or 4 or 5 or 6
25. 7 or 8 or 9 or 10 or 11 or 12 or 13 or 14
26. 15 or 16 or 17 or 18 or 19 or 20
27. 21 and 22 and 23

Web of Science (Web of Science Core Collection)
In title:

((TI=("physical activity" OR exercise OR “sedentary behaviour”)) AND TI=("having a child" OR "having children" OR childbearing OR preconception OR pregnancy OR postpartum OR post-partum OR parenthood OR motherhood OR fatherhood)) AND TI=(england OR "northern ireland" OR scotland OR wales OR "united kingdom" OR uk)

In abstract:

((AB=("physical activity" OR exercise OR “sedentary behaviour”)) AND AB=("having a child" OR "having children" OR childbearing OR preconception OR pregnancy OR postpartum OR post-partum OR parenthood OR motherhood OR fatherhood)) AND AB=(england OR "northern ireland" OR scotland OR wales OR "united kingdom" OR uk)

GREY LITERATURE SEARCH STRATEGIES

Google Scholar
allintitle: ("physical activity" OR exercise OR “sedentary behaviour”) AND ("having a child" OR "having children" OR childbearing OR preconception OR pregnancy OR postpartum OR post-partum OR parenthood OR motherhood OR fatherhood) AND england

allintitle:("physical activity" OR exercise OR “sedentary behaviour”), ("having a child" OR "having children" OR childbearing OR preconception OR pregnancy OR postpartum OR post-partum OR parenthood OR motherhood OR fatherhood) AND "northern ireland"

allintitle: ("physical activity" OR exercise OR “sedentary behaviour”) AND ("having a child" OR "having children" OR childbearing OR preconception OR pregnancy OR postpartum OR post-partum OR parenthood OR motherhood OR fatherhood) AND Scotland

allintitle: ("physical activity" OR exercise OR “sedentary behaviour”) AND ("having a child" OR "having children" OR childbearing OR preconception OR pregnancy OR postpartum OR post-partum OR parenthood OR motherhood OR fatherhood) AND wales

allintitle: ("physical activity" OR exercise OR “sedentary behaviour”) AND ("having a child" OR "having children" OR childbearing OR preconception OR pregnancy OR postpartum OR post-partum OR parenthood OR motherhood OR fatherhood) AND "united kingdom"

allintitle: ("physical activity" OR exercise OR “sedentary behaviour”) AND ("having a child" OR "having children" OR childbearing OR preconception OR pregnancy OR postpartum OR post-partum OR parenthood OR motherhood OR fatherhood) AND uk

ProQuest
In title:

ti("physical activity" OR exercise OR “sedentary behaviour”) AND ti("having a child" OR "having children" OR childbearing OR preconception OR pregnancy OR postpartum OR post-partum OR

For peer review only

parenthood OR motherhood OR fatherhood) AND ti(england OR "northern ireland" OR scotland OR wales OR "united kingdom" OR uk)

In abstract:

ab("physical activity" OR exercise OR “sedentary behaviour”) AND ab("having a child" OR "having children" OR childbearing OR preconception OR pregnancy OR postpartum OR post-partum OR parenthood OR motherhood OR fatherhood) AND ab(england OR "northern ireland" OR scotland OR wales OR "united kingdom" OR uk)

Websites
For example, “Best Beginning’s” and “Tommy’s”.

For peer review only

BMJ Open

The impact of having a child on physical activity in the United Kingdom: A scoping review protocol

Journal:	*BMJ Open*
Manuscript ID	bmjopen-2022-063410.R2
Article Type:	Protocol
Date Submitted by the Author:	30-Aug-2022
Complete List of Authors:	Northcote, Matthew; University of Bristol, School for Policy Studies<br>Foster, Charlie ; University of Bristol, School for Policy Studies<br>Pulsford, Richard; University of Exeter, College of Life and Environmental Sciences<br>Spotswood, Fiona; University of Bristol, School of Management
**Primary Subject Heading**:	Public health
Secondary Subject Heading:	Health policy
Keywords:	PUBLIC HEALTH, SPORTS MEDICINE, SOCIAL MEDICINE

SCHOLARONE™
Manuscripts

THE IMPACT OF HAVING A CHILD ON PHYSICAL ACTIVITY IN THE UNITED KINGDOM: A SCOPING REVIEW PROTOCOL

Matthew Northcote[1], Charlie Foster[1], Richard Pulsford[2], Fiona Spotswood[3]

[1]School for Policy Studies, University of Bristol, Bristol, United Kingdom; [2]College of Life and Environmental Sciences, University of Exeter, Exeter, United Kingdom; [3]School of Management, University of Bristol, Bristol, United Kingdom

Word count: 3,723 (excluding title page, abstract, strengths and limitations of this study, keywords, acknowledgements, contributors, competing interests, funding, and references)

Corresponding author: Matthew Northcote (12 Woodland Road, City of Bristol, Bristol, BS8 1UQ; av20205@bristol.ac.uk)

For peer review only

ABSTRACT

Introduction

Throughout the life course, there are major life transitions that are associated with reduced physical activity, which may have further implications for health and wellbeing. Having a child is one such transition that has been identified as a critical transformative experience and window for intervention. We will conduct a scoping review of available evidence exploring the impact of having a child on physical activity in the United Kingdom.

Methods and analysis

We will use best-practice methodological frameworks to map key concepts and available evidence, summarise and disseminate findings to stakeholders, and identify knowledge gaps. A three-step search strategy will identify primary research studies, including reviews, from published and grey literature, exploring the impact of having a child on physical activity in the United Kingdom, from the preconception period, throughout pregnancy, the postpartum period, and into parenthood. An initial limited search will identify relevant reviews, from which keywords and index terms will be extracted. We will conduct searches of CINAHL, Embase, Medline, PsycINFO, and Web of Science to identify relevant articles written in English from inception to February 2022. Two reviewers will independently screen titles and abstracts of identified studies for inclusion and chart data, with a third reviewer resolving any conflicts. Backwards citation tracking will identify any additional studies. We will conduct numeric and thematic analysis to map data in tabular and diagrammatic format and provide a description of findings by theme.

Ethics and dissemination

Ethical approval is not required for this scoping review. We will disseminate findings to stakeholders through publications, conferences, social media platforms, and in-person communications. Consultations with key stakeholders, with their unique expertise and perspectives, will provide greater insight. We will establish the main priorities for future research to inform the research questions of subsequent studies.

Scoping review registration

Open Science Framework https://osf.io/gtqa4/ DOI 10.17605/OSF.IO/GTQA4

STRENGTHS AND LIMITATIONS OF THIS STUDY

- We will use a scoping review methodology to provide an overview of available evidence exploring the impact of having a child on physical activity in the United Kingdom.
- Scoping reviews are a form of literature review which will allow us to map key concepts and available evidence, exploring the extent, range, and nature of existing evidence.
- These methods will allow us to use broad research questions to define and clarify existing concepts, summarise and disseminate findings to stakeholders, identify knowledge gaps, and determine the main priorities for future research.
- We will use best-practice methodological frameworks to guide the conduct of this scoping review and established guidelines to inform reporting.
- It is important to note that scoping reviews do not address more focussed research questions, like in systematic reviews, nor do they generally include formal critical appraisal of individual sources of evidence using tools.

KEYWORDS

Childbearing; England; Exercise; Fatherhood; Having a child; Having children; Motherhood; Northern Ireland; Parenthood; Physical activity; Postpartum; Post-partum; Preconception; Pregnancy; Scotland; Sedentary behaviour; UK; United Kingdom; Wales

For peer review only - http://bmjopen.bmj.com/site/about/guidelines.xhtml

For peer review only

INTRODUCTION

Physical activity (PA) can be defined as 'any bodily movement produced by skeletal muscles that results in energy expenditure' [1]. The benefits of regular PA participation as a key component of physical, mental, and social health and wellbeing has been well-established [2]. For example, PA can reduce the risk of disease, manage existing conditions, and develop and maintain physical, mental, and social function [3].

Recent evidence from national health surveys in the United Kingdom (UK) indicate that a large proportion of adults (aged 16 and over) are less physically active than the current UK Chief Medical Officer's PA guidelines recommend [3]. Specifically, 34% in England in 2016 [4]; 45% in Northern Ireland between 2016 and 2017 [5]; 54% in Scotland in 2020 [6]; and 44% in Wales between 2020 and 2021 [7] did not meet PA guidelines for aerobic activity (i.e., at least 150 minutes of moderate-intensity activity, or 75 minutes of vigorous-intensity activity, or shorter durations of very-vigorous-intensity activity, or combination of moderate-, vigorous-, and very-vigorous-intensity activity each week). It is necessary here to note the likely negative impact of lockdown measures to curb the spread of the 2019 coronavirus in surveys conducted between 2020 and 2021. Moreover, of those who met recommendations for aerobic activity, over a third, 35% in England in 2016 [4] and 37% in Scotland in 2019 [8], did not meet that for muscle-strengthening activity (i.e., at least two or more days each week).

According to PA guidelines, adults are to minimise the amount of time spent being sedentary and break up long periods of inactivity with at least light-intensity activity [3]. Sedentary behaviour (SB) can be defined as 'any waking behaviour characterized by an energy expenditure ≤1.5 [metabolic equivalents of task (METs)] while in a sitting or reclining posture' [9]. In adults, high volumes of SB heighten the risk of all-cause mortality, cardiovascular mortality, cardiovascular disease incidence, and type-2 diabetes incidence [2,10-12]. In England, men are more likely than women to have increased time spent being sedentary during both the weekday (4.9 hours and 4.7 hours, respectively) and weekend (5.4 hours and 5.1 hours, respectively) [13].

Although physical inactivity and SB are distinct constructs [14], PA, including light-intensity (i.e., 1.6 to 2.9 METs), moderate-intensity (i.e., 3 to 5.9 METs), and vigorous-intensity (≥6 METs) activities, and SB exist on the same energy expenditure spectrum [15]. In fact, if we assume adults sleep an average of eight hours each night, only around 2% of our waking time is required to meet PA guidelines for aerobic activity in the UK [3]. Meanwhile, the remaining 98% of our waking time is spent in SB and light-intensity activity, including static (e.g., standing) and ambulatory activities [14]. As a result, it is important to explore activities across the energy expenditure spectrum, not merely the least practiced moderate-to-vigorous-intensity physical activity (MVPA). Furthermore, PA participation modifies the effect of SB on mortality, where higher amounts of MVPA can mitigate all-cause, cardiovascular disease, and cancer related deaths [2,10,16]. For example, high levels of moderate-intensity activity (i.e., 60 to 75 minutes per day) has been found to eliminate the risk of mortality associated with high sitting time, whilst only attenuating the risk associated with high television-viewing time [16].

In the UK, physical inactivity accounts for one in six deaths and has an estimated annual cost of £7.4 billion, including £0.9 billion to the National Health Service (NHS) alone [17]. Moreover, prolonged SB (≥6 hours each day) was estimated to cost the NHS £0.8 billion between 2016 and 2017, which, if eliminated, could have avoided 69,276 deaths [18]. Considering the prevalence and economic and social burden of physical inactivity and SB, it is important to identify populations that are less physically active and/or more sedentary and characterise and understand these contexts to develop targeted interventions which promote PA.

Throughout the life course, there are major life events and transitions that are associated with reduced PA, which may have further implications for health and wellbeing [19]. Life events 'mark the beginning or end of a specific status. A status is a nominal variable with at least two levels' [20]. For

For peer review only - http://bmjopen.bmj.com/site/about/guidelines.xhtml

example, the life event of parenthood, or having a child, could be conceptualised to involve multiple consecutive shifts in status from preconception (or non-parent) to pregnant, postpartum (until six months after giving birth [21]), then finally into parenthood (or parent). Notably, life events can bring about periods of biological, psychological, and/or social adaption and readjustment which can subsequently lead to behaviour change, such as reduced PA participation [22]. Life transitions are conceptualised through these adaptive periods and reflect the processual character and temporality of life events, including their anticipation (to the extent that they can be anticipated), history, and eventual aftermath [22]. As a result, both life events and transitions are inherently interconnected yet differ in their temporal characteristics. For instance, life events are singular occurrences which mark a change in status, whilst life transitions represent the duration of that given status change [20,22]. These conceptualisations of life events and transitions exclude minor life events, such as daily hassles; slow transitions without clearly identifiable life events, like ageing; and non-events, such as not having a child [20,22].

Parenthood, or having a child, is one such life event and transition that has been identified as a critical transformative experience and window for adult health and wellbeing and intervention [23]. Moreover, having a child is often interconnected with other life events and transitions, including changes to relationship status, and living situation, which may further compound these effects [24]. As a result, the impact of having a child on PA should be characterised and understood in its entirety, from the preconception period, throughout pregnancy, the postpartum period, and beyond into parenthood, and contextually among other related life events and transitions.

The latest reviews of existing evidence exploring the impact of having a child on PA by Bellows-Riecken and Rhodes [25] and Corder et al. [26] suggest that the transition into parenthood, becoming a parent, generally reduces PA participation, particularly in new mothers. Although both reviews provide valuable characterisations and understandings of PA during this time, their scope focusses on the differences between parents and non-parents and does not consider the other relevant changes in status embedded in the 'having' of a child, such as becoming pregnant and entering the postpartum period, with their inherent temporal and processual characteristics. In addition, although both reviews include quantitative and qualitative evidence, these were only sourced from published literature, consequently missing the potentially rich insights afforded by grey literature. Both reviews also chose to forgo the characterisation and understanding of the relationship between PA and SB. What's more, these reviews included international sources of evidence from a variety of countries, namely the United States, Australia, Canada, and the UK, among others from Europe. Whilst these findings may be generalisable to other western countries, it is important to ensure findings are culturally and contextually relevant to the UK, making them more applicable and practicable within this setting. Given that there were 585,195 live births in England in 2020 [27], it is important to provide an overview of available evidence exploring the impact of having a child in the UK.

Recent evidence exploring the impact of having a child on PA by intensity, including SB, indicates that parents participate in significantly more light-intensity activity but less MVPA and SB, compared to those without children [28-29]. For example, in the study by Gaston et al. [28], mothers took part in more light-intensity activity ($p = 0.001$) but less MVPA ($p = 0.000$) than women without children. Meanwhile, fathers only engaged in more light-intensity activity, compared to men without children ($p = 0.000$). These associations differed by the number and age of children. For instance, mothers with one child, new mothers, participated in more light-intensity activity ($p = 0.005$) than women without children, while mothers with two or three or more children took part in less MVPA ($p = 0.00$ and $p = 0.005$, respectively). Notably, mothers whose youngest child was aged 12 to 15 engaged in more SB ($p = 0.017$), compared to women without children, whilst mothers whose youngest child was aged less than six participated in more light-intensity activity ($p = 0.000$) and less MVPA ($p = 0.000$). In fathers, those with two children took part in more light-intensity activity than men without children ($p = 0.000$). Furthermore, fathers whose youngest child was aged less than six engaged in more light-intensity activity ($p = 0.000$) but less MVPA ($p = 0.000$), compared to men without children. Considering

For peer review only - http://bmjopen.bmj.com/site/about/guidelines.xhtml

that parents participate in more light-intensity activity and less MVPA and SB than those without children, it is necessary to review available evidence exploring PA by intensity and examine the relationship between PA and SB across the entirety of having a child, from the preconception period, throughout pregnancy, the postpartum period, and into parenthood.

Preliminary searches broadened our knowledge of available evidence and informed the formulation of research questions. We conducted searches of Medline and Google Scholar to identify reviews exploring the impact of having a child on PA in the UK. The search terms were 'having a child' AND 'physical activity' AND 'review' AND ('United Kingdom' OR 'UK'). No relevant reviews were identified. We conducted further searches to identify relevant primary research studies by removing the search term relating to reviews. No studies were identified from Medline. Only one study was identified as relevant from the first 100 of 3,080 results from Google Scholar [30]. This study by Werneck et al. [30] examined the association between family-related life-events (cohabitation/marriage and becoming a parent) and change in PA in the UK. Findings from this study suggest that becoming a parent was associated with a decline in PA among men ($\beta = -0.234$, 95% CI -0.396 to -0.072), but not women ($\beta = 0.126$, 95% CI -0.048 to 0.301). Specifically, men who became fathers while cohabiting ($\beta = -0.201$, 95% CI -0.383 to -0.020) and without cohabiting ($\beta = -0.937$, 95% CI -1.623 to -0.250) experienced greater declines in PA compared to those remaining single and without children, where non-cohabiting fathers experienced the greatest decline. These associations did not differ by the age of children. Since no relevant reviews were identified and a dearth of primary research studies, we aim to provide an overview of available evidence exploring the impact of having a child on PA in the UK.

METHODS AND ANALYSIS

We will conduct a review of available evidence exploring the impact of having a child on PA in the UK using best-practice scoping review methodologies. Although there is no consensus definition for scoping reviews [31-32], it is a form of literature review which can map key concepts and available evidence within a given topic area, exploring the extent, range, and nature of existing evidence [33-34]. Moreover, scoping reviews can define and clarify existing concepts, summarise and disseminate findings to stakeholders, such as practitioners and policymakers, identify knowledge gaps, and determine the main priorities for future research [33-34].

A scoping review, rather than a systematic review, is more congruent with the aims and purposes of this study. Specifically, scoping reviews use broad research questions to provide an overview of the available evidence [33]. Meanwhile, systematic reviews use focussed research questions to identify the best evidence. Despite being useful as a precursor to systematic reviews, scoping reviews are legitimate in their own right as standalone studies and are not required to be followed up with a systematic review [33]. Furthermore, although scoping reviews are not, by name, systematic, they do follow a rigorous systematic process [35-36]. Unlike systematic reviews, it is important to recognise that best-practice methodological frameworks for scoping reviews do not recommend critical appraisal of individual sources of evidence, although this is still possible with reasonable justification [35-38]. Plainly, scoping reviews should not be considered a rapid and cheap alternative to systematic reviews, where they map the breadth of available literature and, as such, require a substantial amount of time and resources, including labour and finances [33].

We will use best-practice scoping review methodological frameworks by Arksey and O'Malley [33], adapted by Levac et al. [34] and the Joanna Briggs Institute (JBI) [36-37], to guide the conduct of this review. This is an iterative process by which research questions and methods may be further developed with the emergent evidence. We will report any deviation from this protocol in the final review. In addition, we will use the Preferred Reporting Items for Systematic reviews and Meta-Analysis extension for Scoping Reviews (PRISMA-ScR) to guide the reporting of this protocol as detailed in **supplementary material 1** [39]. Furthermore, methods were informed by previous scoping reviews in PA for health research [22,40-42]. This scoping review is registered with the Open Science Framework (OSF) to enhance the rigorousness and transparency of this research process.

For peer review only - http://bmjopen.bmj.com/site/about/guidelines.xhtml

Patient and public involvement
None.

Stage 1: Identifying the research questions
The following broad research questions were informed by preliminary searches and interdisciplinary discussions within the research team. Moreover, research questions were formulated using the P.C.C. (Population, Concept, Context) mnemonic which will help to identify relevant studies and establish appropriate eligibility criteria [37].

- What is known about the impact of having a child on PA in the UK?
    - What is known about the relationship between PA and SB in this field?
- What are the knowledge gaps in this field?
- What are the main priorities for future research in this field?

Stage 2: Identifying relevant studies
Eligibility criteria
The following a priori eligibility criteria was informed by preliminary searches, interdisciplinary discussions within the research team, and research questions.

- Participants have to be adults that are reported to have been planning to have (i.e., preconception, non-parents), having (i.e., pregnant), have had (i.e., postpartum and into parenthood, parents), or planned to have (i.e., stillbirth) a child.
- Participants can be from any group, including general and specific populations (e.g., those recruited based on their living with a physical or mental illness or condition).
- Participants can be of any sex, ethnicity, parity, relationship status, and socio-economic position [43].
- Findings must explore the impact of having a child on PA, but not the impact of PA on health and wellbeing.
- Findings must report data on PA and/or SB which was assessed either quantitatively (i.e., device-derived or through self-report) and/or qualitatively (e.g., attitudes, views, perspectives, experiences, etc.).
- Studies can be published literature (e.g., primary research studies, including reviews) or grey literature (e.g., unpublished dissertations/theses or conference proceedings).
- Studies must be of quantitative observational (e.g., cross-sectional, prospective cohort), qualitative, or mixed methods study design.
- Studies must not have an experimental study design (e.g., randomised controlled trials [RCTs]) evaluating adherence and/or acceptability of interventions, or secondary analysis thereof; guidelines, opinion pieces (e.g., commentaries or editorials); or popular press articles (e.g., magazine or newspaper articles).
- Studies must be conducted with data generated exclusively within the UK, including England, Northern Ireland, Scotland, and Wales.
- Studies must be written in the English language.

Search strategy
Step 1: An initial limited search
We conducted an initial limited search of Medline (Ovid) and Google Scholar (Advanced Search) for relevant reviews. Given that there were no reviews exploring the impact of having a child on PA in the UK, we expanded search terms to allow for those using data generated elsewhere, including 'having a child' AND 'physical activity' AND 'review'. The two records identified from Medline and the first 100 of the 6,720 records identified from Google Scholar were reviewed. A total of five reviews were identified as relevant [22,25,44-46]. We also conducted a search of ProQuest to identify any similar dissertations but did not retrieve any records.

For peer review only - http://bmjopen.bmj.com/site/about/guidelines.xhtml

Step 2: Identifying keywords and search terms

We used keywords and index terms featured within the titles and abstracts of the relevant reviews to inform main searches. Main searches will identify studies exploring the impact of having a child on PA in the UK, from the preconception period, throughout pregnancy, the postpartum period, and into parenthood, from inception to February 2022. We first prepared an a priori search strategy for published literature within the Medline electronic bibliographic database, with Boolean operators and Medical Subject Headings (i.e., MeSH terms). We then adapted this search strategy for searches within CINAHL (EBSCOhost), Embase (Ovid), PsycINFO (Ovid), and Web of Science databases (Clarivate). All search strategies are detailed in **supplementary material 2**. Search terms will relate to those status shifts involved in 'having a child' in an attempt to remain inclusive, where having a child can impact a variety of populations, including those of differing sex, ethnicity, parity, relationship status, and socio-economic position [43]. Moreover, search terms will relate to the main concept of 'physical activity', including 'sedentary behaviour', and the context of the 'United Kingdom'. We have made a pragmatic decision to only search for studies which have these search terms in the title and/or abstract due to the practical constraints of this review.

We will use a similar search strategy for grey literature, where the search terms used for Medline has been adapted for Google Scholar and ProQuest (**supplementary material 2**). In Google Scholar, this will involve a reduced set of search terms and multiple searches due to the limitation on characters. Moreover, in Google Scholar, it is only possible to search for studies which have these search terms in the title. We will also search websites of organisations (e.g., Best Beginning's, Tommy's) involved in the commissioning, undertaking, or cataloguing of research in this field and, if appropriate, we will make contact by email.

Step 3: Further searching of references and citations

We will conduct backwards citation tracking of included studies to identify further potentially eligible studies. Considering that scoping reviews are typically iterative, additional search terms and information sources (e.g., electronic bibliographic databases and websites) may be added to existing search strategies at any time throughout the research process. We may also contact corresponding authors of included studies to request further information. We will make search strategies available from the OSF registration. We will import references of identified studies into Endnote X9.2 reference management software and organise them by database of origin, with duplicates removed.

Stage 3: Study selection

Titles and abstracts of identified studies will be predominantly screened for eligibility by one researcher (MN). A second researcher (JB) will independently complete this same process on a random sample of 10% of titles and abstracts. If the same screening decisions are taken by both researchers for 95% of studies, then MN will screen the remaining titles and abstracts. Meanwhile, if the same decisions are taken for less than 95% of studies, then MN and JB will meet to discuss these decisions with reference to the eligibility criteria and repeat this process. Any conflicts after this point will be resolved by a third researcher (CF/RP). If there is no consensus between these three parties, then we will include the study in the review. All researchers will meet to discuss the eligibility criteria before embarking on this process to minimise these occurrences.

Stage 4: Charting the data

Data charting of included studies will be predominantly conducted by MN. A data charting form will be employed, using the a priori headings detailed below [37]. Given that scoping reviews are typically iterative, additional headings may be added to the form throughout the research process. The data charting process will involve MN charting 90% of studies and JB charting 10% of studies, with JB checking 10% of MN's data and vice versa. If there are considerable differences between researchers, MN and JB will meet to discuss decisions with reference to the data charting form and rectify any issues. Any conflicts will be resolved by CF/RP during group meetings. If a full text article cannot be

For peer review only - http://bmjopen.bmj.com/site/about/guidelines.xhtml

retrieved, MN will attempt to gain access through the university library and by contacting corresponding authors. If the full text article is not accessible, the study will be excluded.

- Author(s)
- Year of publication
- Source and country of origin
- Aims/purpose
- Study population and sample size (if applicable)
- Methodology/methods
- Concept
- Findings and details of these (e.g., how measured) (if applicable)
- Key findings by theme that relate to research questions

Stage 5: Collating, summarising, and reporting the findings

We will conduct a numeric analysis of included studies to describe review decisions, findings from searches, removal of duplicates, study selection, full text retrieval, and backwards citation tracking [37]. We will present these findings using a study flow diagram. In addition, we will map data in a tabular and diagrammatic format by period of publication, country of origin, study type, and themes. Considering that scoping reviews are typically an iterative process, we may include additional categories of data. We will also conduct a thematic analysis of included studies using NVivo 12 qualitative data analysis software to provide a description of key concepts relevant to research questions and findings by theme. We will analyse these findings whilst being open to socio-demographic differences in the target population, such as sex, ethnicity, parity, relationship status, and socio-economic position [43]. Furthermore, we will use the PRISMA-ScR framework to guide the reporting of this study [39].

Critical appraisal of individual sources of evidence

We will not conduct critical appraisal of individual sources of evidence, where this is consistent with methodological frameworks for scoping reviews [33].

ETHICS AND DISSEMINATION

Ethical approval will not be required for this scoping review. This review will provide a novel insight into the impact of having a child on PA in the UK. Specifically, we will map key concepts and available evidence, identify knowledge gaps, and determine the main priorities for future research. We will subsequently summarise and submit findings for peer review and publication in a relevant open-access journal. Moreover, we will develop an infographic to be disseminated at conferences and online via professional social media channels, such as Twitter. Importantly, findings from this study will be reported regardless of the nature of the relationships between having a child and PA in the UK. As previously stated, we also aim to consult with key stakeholders, including practitioners and policymakers, to comment on findings and provide greater insight with their unique expertise and perspectives [34,47].

ACKNOWLEDGEMENTS

The authors would like to thank Dr Joey Murphy for his guidance in identifying relevant literature pertaining to best-practice methodological frameworks for scoping reviews. We would also like to thank Jack Brazier (JB), a non-author, for accepting the role of second researcher within the review selection and data charting processes.

CONTRIBUTORS

MN, CF, RP, and FS formulated the research questions for this scoping review. CF identified the methodological frameworks and methods. MN prepared the methods. MN, CF, and RP contributed to

For peer review only - http://bmjopen.bmj.com/site/about/guidelines.xhtml

the development of the search strategy. MN wrote the first and subsequent drafts of the manuscript. All authors reviewed and approved these manuscripts.

COMPETING INTERESTS

None declared.

FUNDING

This study was part of MN's PhD research at the Centre for Exercise, Nutrition, and Health Sciences, School for Policy Studies, University of Bristol. MN is a PhD Researcher with the South West Doctoral Training Partnership (SWDTP) which is funded by the Economic and Social Research Council (ESRC) (grant number: ES/P000630/1).

REFERENCES

1 Capsersen CJ, Powell KE, Christenson GM. Physical activity, exercise, and physical fitness: definitions and distinctions for health-related research. *Public Health Rep* 1985 Mar-Apr;100(2):126–30.

2 2018 Physical Activity Guidelines Advisory Committee. Physical Activity Guidelines Advisory Committee Scientific Report. Washington DC: United States Department of Health and Human Services. 2018.

3 Department of Health and Social Care. UK Chief Medical Officers' Physical Activity Guidelines. 2019.

[dataset] [4] NHS Digital. Data from: Health Survey for England, 2016: Adult physical activity – tables. Health Survey for England, 2016, December 13, 2017.

5 Department of Health. Health Survey (NI) First Results 2016/17. 2017.

[dataset] [6] Scottish Government. Data from: Chapter 5 Tables. Scottish Health Survey – telephone survey – August/September 2020: main report, January 26, 2021.

[dataset] [7] Welsh Government. Data from: Adult lifestyles by local authority and health board, 2020-21 onwards. StatsWales, July 2022.

[dataset] [8] Scottish Government. Data from: Chapter 7 – Physical Activity Tables. Scottish Health Survey 2019 – volume 1: main report, September 29, 2020.

9 Tremblay MS, Aubert S, Barnes JD, Saunders TJ, Carson V, Latimer-Cheung AE, Chastin SF, Altenburg TM, Chinapaw MJ. Sedentary behavior research network (SBRN)–terminology consensus project process and outcome. *Int J Behav Nutr Phys Act* 2017 Jun 10;14(1):75. doi: 10.1186/s12966-017-0525-8

10 Ekelund U, Brown WJ, Steene-Johannessen J, Fagerland MW, Owen N, Powell KE, Bauman AE, Lee IM. Do the associations of sedentary behaviour with cardiovascular disease mortality and cancer mortality differ by physical activity level? A systematic review and harmonised meta-analysis of data from 850 060 participants. *Br J Sports Med* 2019 Jul;53(14):886-894. doi: 10.1136/bjsports-2017-098963

11 Ekelund U, Tarp J, Steene-Johannessen J, Hansen BH, Jefferis B, Fagerland MW, Whincup P, Diaz KM, Hooker SP, Chernofsky A, Larson MG. Dose-response associations between accelerometry measured physical activity and sedentary time and all cause mortality: systematic review and harmonised meta-analysis. *BMJ* 2019 Aug 21;366:l4570. doi: 10.1136/bmj.l4570

12 Patterson R, McNamara E, Tainio M, de Sá TH, Smith AD, Sharp SJ, Edwards P, Woodcock J, Brage S, Wijndaele K. Sedentary behaviour and risk of all-cause, cardiovascular and cancer mortality, and incident type 2 diabetes: a systematic review and dose response meta-analysis. *Eur J Epidemiol* 2018 Sep;33(9):811-829. doi: 10.1007/s10654-018-0380-1

For peer review only - http://bmjopen.bmj.com/site/about/guidelines.xhtml

13 Scholes S, Mindell J. Health Survey for England – 2012: Chapter 2, Physical activity in adults. 2013

14 van der Ploeg HP, Hillsdon M. Is sedentary behaviour just physical inactivity by another name?. Int *J Behav Nutr Phys Act* 2017 Oct 23;14(1):142. doi: 10.1186/s12966-017-0601-0

15 Ainsworth BE, Haskell WL, Herrmann SD, Meckes N, Bassett DR, Tudor-Locke C, Greer JL, Vezina J, Whitt-Glover MC, Leon AS. 2011 Compendium of Physical Activities: a second update of codes and MET values. *Med Sci Sports Exerc* 2011 Aug;43(8):1575-81. doi: 10.1249/MSS.0b013e31821ece12

16 Ekelund U, Steene-Johannessen J, Brown WJ, Fagerland MW, Owen N, Powell KE, Bauman A, Lee IM, Lancet Physical Activity Series 2 Executive Committee, Lancet Sedentary Behaviour Working Group. Does physical activity attenuate, or even eliminate, the detrimental association of sitting time with mortality? A harmonised meta-analysis of data from more than 1 million men and women. *Lancet*. 2016 Sep 24;388(10051):1302-10. doi: 10.1016/S0140-6736(16)30370-1

17 Office for Health Improvement & Disparities. Physical activity: applying All Our Health. 2022.

18 Heron L, O'Neill C, McAneney H, Kee F, Tully MA. Direct healthcare costs of sedentary behaviour in the UK. *J Epidemiol Community Health* 2019 Jul;73(7):625-629. doi: 10.1136/jech-2018-211758

19 Allender S, Hutchinson L, Foster C. Life-change events and participation in physical activity: a systematic review. *Health Promot Int* 2008 Jun;23(2):160-72. doi: 10.1093/heapro/dan012

20 Luhmann M, Hofmann W, Eid M, Lucas RE. Subjective well-being and adaptation to life events: a meta-analysis. *J Pers Soc Psychol* 2012 Mar;102(3):592-615. doi: 10.1037/a0025948

21 Chauhan G, Tadi P. Physiology, Postpartum Changes. Available: https://www.ncbi.nlm.nih.gov/books/NBK555904/ [Accessed 29 Jul 2022].

22 Gropper H, John JM, Sudeck G, Thiel A. The impact of life events and transitions on physical activity: A scoping review. *PLoS One* 2020 Jun 22;15(6):e0234794. doi: 10.1371/journal.pone.0234794

23 Saxbe D, Rossin-Slater M, Goldenberg D. The transition to parenthood as a critical window for adult health. *Am Psychol* 2018 Dec;73(9):1190-1200. doi: 10.1037/amp0000376

24 Hull EE, Rofey DL, Robertson RJ, Nagle EF, Otto AD, Aaron DJ. Influence of marriage and parenthood on physical activity: a 2-year prospective analysis. *J Phys Act Health* 2010 Sep;7(5):577-83. doi: 10.1123/jpah.7.5.577

25 Bellows-Riecken KH, Rhodes RE. A birth of inactivity? A review of physical activity and parenthood. *Prev Med* 2008 Feb;46(2):99-110. doi: 10.1016/j.ypmed.2007.08.003

26 Corder K, Winpenny EM, Foubister C, Guagliano JM, Hartwig XM, Love R, Clifford Astbury C, van Sluijs EM. Becoming a parent: A systematic review and meta-analysis of changes in BMI, diet, and physical activity. *Obes Rev* 2020 Apr;21(4):e12959. doi: 10.1111/obr.12959

[dataset] [27] Office for National Statistics. Data from: Births in England and Wales: summary tables. October 14, 2021.

28 Gaston A, Edwards SA, Doelman A, Tober JA. The impact of parenthood on Canadians' objectively measured physical activity: an examination of cross-sectional population-based data. *BMC Public Health.* 2014 Nov 3;14:1127. doi: 10.1186/1471-2458-14-1127

29 Rhodes RE, Blanchard CM, Benoit C, Levy-Milne R, Naylor PJ, Symons Downs D, Warburton DE. Physical activity and sedentary behavior across 12 months in cohort samples of couples without children, expecting their first child, and expecting their second child. *J Behav Med* 2014 Jun;37(3):533-42. doi: 10.1007/s10865-013-9508-7

For peer review only - http://bmjopen.bmj.com/site/about/guidelines.xhtml

30 Werneck AO, Winpenny EM, Van Sluijs EM, Corder K. Cohabiting and becoming a parent: associations with changes in physical activity in the 1970 British cohort study. *BMC Public Health* 2020 Jul 10;20(1):1085. doi: 10.1186/s12889-020-09187-2

31 Colquhoun HL, Levac D, O'Brien KK, Straus S, Tricco AC, Perrier L, Kastner M, Moher D. Scoping reviews: time for clarity in definition, methods, and reporting. *J Clin Epidemiol* 2014 Dec;67(12):1291-4. doi: 10.1016/j.jclinepi.2014.03.013

32 Peterson J, Pearce PF, Ferguson LA, Langford CA. Understanding scoping reviews: Definition, purpose, and process. *J Am Assoc Nurse Pract* 2017 Jan;29(1):12-6. doi: 10.1002/2327-6924.12380

33 Arksey H, O'Malley L. Scoping studies: towards a methodological framework. *Int J Soc Res Methodol*. 2005 Feb;8(1):19-32. doi: 10.1080/1364557032000119616

34 Levac D, Colquhoun H, O'Brien KK. Scoping studies: advancing the methodology. *Implement Sci*. 2010 Sep 20;5:69. doi: 10.1186/1748-5908-5-69

35 Polit DF, Beck CT. Systematic reviews of research evidence: Meta-analysis, metasynthesis, and mixed studies review. In: Polit DF, Beck CT, 9th ed. Nursing research: Generating and assessing evidence for nursing practice. Philadeplhia, PA. 2012.

36 Peters MD, Marnie C, Tricco AC, Pollock D, Munn Z, Alexander L, McInerney P, Godfrey CM, Khalil H. Updated methodological guidance for the conduct of scoping reviews. *JBI Evid Synth* 2020 Oct;18(10):2119-2126. doi: 10.11124/JBIES-20-00167

37 Peters MD, Godfrey CM, Khalil H, McInerney P, Parker D, Soares CB. Guidance for conducting systematic scoping reviews. *Int J Evid Based Healthc* 2015 Sep;13(3):141-6. doi: 10.1097/XEB.0000000000000050

38 Khalil H, Peters M, Godfrey CM, McInerney P, Soares CB, Parker D. An evidence-based approach to scoping reviews. *Worldviews Evid Based Nurs*. 2016 Apr;13(2):118-23. doi: 10.1111/wvn.12144

39 Tricco AC, Lillie E, Zarin W, O'Brien KK, Colquhoun H, Levac D, Moher D, Peters MD, Horsley T, Weeks L, Hempel S. PRISMA extension for scoping reviews (PRISMA-ScR): checklist and explanation. *Ann Intern Med* 2018 Oct 2;169(7):467-473. doi: 10.7326/M18-0850

40 Murray AD, Daines L, Archibald D, Hawkes RA, Schiphorst C, Kelly P, Grant L, Mutrie N. The relationships between golf and health: a scoping review. *Br J Sports Med* 2017 Jan;51(1):12-19. doi: 10.1136/bjsports-2016-096625

41 Griffin SA, Perera NK, Murray A, Hartley C, Fawkner SG, Kemp SP, Stokes KA, Kelly P. The relationships between rugby union, and health and well-being: a scoping review. *Br J Sports Med* 2021 Mar;55(6):319-326. doi: 10.1136/bjsports-2020-102085

42 Williamson C, Baker G, Mutrie N, Niven A, Kelly P. Get the message? A scoping review of physical activity messaging. *Int J Behav Nutr Phys Act* 2020 Apr 15;17(1):51. doi: 10.1186/s12966-020-00954-3

43 Galobardes B, Lynch J, Smith GD. Measuring socioeconomic position in health research. *Br Med Bull* 2007;81-82:21-37. doi: 10.1093/bmb/ldm001

44 Engberg E, Alen M, Kukkonen-Harjula K, Peltonen JE, Tikkanen HO, Pekkarinen H. Life events and change in leisure time physical activity. *Sports Med* 2012 May 1;42(5):433-47. doi: 10.2165/11597610-000000000-00000

45 Condello G, Puggina A, Aleksovska K, Buck C, Burns C, Cardon G, Carlin A, Simon C, Ciarapica D, Coppinger T, Cortis C. Behavioral determinants of physical activity across the life course: a “DEterminants of DIet and Physical ACtivity”(DEDIPAC) umbrella systematic literature review. *Int J Behav Nutr Phys Act* 2017 May 2;14(1):58. doi: 10.1186/s12966-017-0510-2

For peer review only - http://bmjopen.bmj.com/site/about/guidelines.xhtml

46 Stults-Kolehmainen MA, Sinha R. The effects of stress on physical activity and exercise. *Sports Med* 2014 Jan;44(1):81-121. doi: 10.1007/s40279-013-0090-5

47 Pham MT, Rajić A, Greig JD, Sargeant JM, Papadopoulos A, McEwen SA. A scoping review of scoping reviews: advancing the approach and enhancing the consistency. *Res Synth Methods* 2014 Dec;5(4):371-85. doi: 10.1002/jrsm.1123

For peer review only

For peer review only - http://bmjopen.bmj.com/site/about/guidelines.xhtml

Preferred Reporting Items for Systematic reviews and Meta-Analyses extension for Scoping Reviews (PRISMA-ScR) Checklist

SECTION	ITEM	PRISMA-ScR CHECKLIST ITEM	REPORTED ON PAGE #
**TITLE**			
Title	1	Identify the report as a scoping review.	Title page
**ABSTRACT**			
Structured summary	2	Provide a structured summary that includes (as applicable): background, objectives, eligibility criteria, sources of evidence, charting methods, results, and conclusions that relate to the review questions and objectives.	1
**INTRODUCTION**			
Rationale	3	Describe the rationale for the review in the context of what is already known. Explain why the review questions/objectives lend themselves to a scoping review approach.	2-4
Objectives	4	Provide an explicit statement of the questions and objectives being addressed with reference to their key elements (e.g., population or participants, concepts, and context) or other relevant key elements used to conceptualize the review questions and/or objectives.	4-5
**METHODS**			
Protocol and registration	5	Indicate whether a review protocol exists; state if and where it can be accessed (e.g., a Web address); and if available, provide registration information, including the registration number.	1
Eligibility criteria	6	Specify characteristics of the sources of evidence used as eligibility criteria (e.g., years considered, language, and publication status), and provide a rationale.	5
Information sources*	7	Describe all information sources in the search (e.g., databases with dates of coverage and contact with authors to identify additional sources), as well as the date the most recent search was executed.	5-6
Search	8	Present the full electronic search strategy for at least 1 database, including any limits used, such that it could be repeated.	6, supplementary material 2
Selection of sources of evidence†	9	State the process for selecting sources of evidence (i.e., screening and eligibility) included in the scoping review.	6
Data charting process‡	10	Describe the methods of charting data from the included sources of evidence (e.g., calibrated forms or forms that have been tested by the team before their use, and whether data charting was done independently or in duplicate) and any processes for obtaining and confirming data from investigators.	6-7
Data items	11	List and define all variables for which data were sought and any assumptions and simplifications made.	5, 7
Critical appraisal of individual	12	If done, provide a rationale for conducting a critical appraisal of included sources of evidence; describe	7

For peer review only

SECTION	ITEM	PRISMA-ScR CHECKLIST ITEM	REPORTED ON PAGE #
sources of evidence§		the methods used and how this information was used in any data synthesis (if appropriate).	
Synthesis of results	13	Describe the methods of handling and summarizing the data that were charted.	7
**RESULTS**			
Selection of sources of evidence	14	Give numbers of sources of evidence screened, assessed for eligibility, and included in the review, with reasons for exclusions at each stage, ideally using a flow diagram.	NA
Characteristics of sources of evidence	15	For each source of evidence, present characteristics for which data were charted and provide the citations.	NA
Critical appraisal within sources of evidence	16	If done, present data on critical appraisal of included sources of evidence (see item 12).	NA
Results of individual sources of evidence	17	For each included source of evidence, present the relevant data that were charted that relate to the review questions and objectives.	NA
Synthesis of results	18	Summarize and/or present the charting results as they relate to the review questions and objectives.	NA
**DISCUSSION**			
Summary of evidence	19	Summarize the main results (including an overview of concepts, themes, and types of evidence available), link to the review questions and objectives, and consider the relevance to key groups.	NA
Limitations	20	Discuss the limitations of the scoping review process.	1,4
Conclusions	21	Provide a general interpretation of the results with respect to the review questions and objectives, as well as potential implications and/or next steps.	NA
**FUNDING**			
Funding	22	Describe sources of funding for the included sources of evidence, as well as sources of funding for the scoping review. Describe the role of the funders of the scoping review.	8

JBI = Joanna Briggs Institute; PRISMA-ScR = Preferred Reporting Items for Systematic reviews and Meta-Analyses extension for Scoping Reviews.

* Where *sources of evidence* (see second footnote) are compiled from, such as bibliographic databases, social media platforms, and Web sites.

† A more inclusive/heterogeneous term used to account for the different types of evidence or data sources (e.g., quantitative and/or qualitative research, expert opinion, and policy documents) that may be eligible in a scoping review as opposed to only studies. This is not to be confused with *information sources* (see first footnote).

‡ The frameworks by Arksey and O'Malley (6) and Levac and colleagues (7) and the JBI guidance (4, 5) refer to the process of data extraction in a scoping review as data charting.

§ The process of systematically examining research evidence to assess its validity, results, and relevance before using it to inform a decision. This term is used for items 12 and 19 instead of "risk of bias" (which is more applicable to systematic reviews of interventions) to include and acknowledge the various sources of evidence that may be used in a scoping review (e.g., quantitative and/or qualitative research, expert opinion, and policy document).

From: Tricco AC, Lillie E, Zarin W, O'Brien KK, Colquhoun H, Levac D, et al. PRISMA Extension for Scoping Reviews (PRISMAScR): Checklist and Explanation. Ann Intern Med. 2018;169:467–473. doi: 10.7326/M18-0850.

For peer review only - http://bmjopen.bmj.com/site/about/guidelines.xhtml

SUPPLEMENTARY MATERIAL 2: SEARCH STRATEGIES

PUBLISHED LITERATURE SEARCH STRATEGIES

CINAHL (EBSCOhost)

In title:

("Physical activity" OR Exercise OR "Sedentary behaviour") AND ("Having a child" OR "Having children" OR Childbearing OR Preconception OR Pregnancy OR Postpartum OR Post-partum OR Parenthood OR Motherhood OR Fatherhood) AND (England OR "Northern Ireland" OR Scotland OR Wales OR "United Kingdom" OR UK)

In abstract:

("Physical activity" OR Exercise OR "Sedentary behaviour") AND ("Having a child" OR "Having children" OR Childbearing OR Preconception OR Pregnancy OR Postpartum OR Post-partum OR Parenthood OR Motherhood OR Fatherhood) AND (England OR "Northern Ireland" OR Scotland OR Wales OR "United Kingdom" OR UK)

Embase (Embase 1974 to 2022 February 04)

1. Physical activity.ti,ab.
2. Physical activity/
3. Exercise.ti,ab.
4. Exercise/
5. Sedentary behaviour.ti,ab.
6. Having a child.ti,ab.
7. Having children.ti,ab.
8. Childbearing.ti,ab.
9. Preconception.ti,ab.
10. Pregnancy.ti,ab.
11. Pregnancy/
12. Postpartum.ti,ab.
13. Post-partum.ti,ab.
14. Parenthood.ti,ab.
15. Parenthood/
16. Motherhood.ti,ab.
17. Fatherhood.ti,ab.
18. England.ti,ab.
19. England/
20. Northern Ireland.ti,ab.
21. Northern Ireland/
22. Scotland.ti,ab.
23. Scotland/
24. Wales.ti,ab.
25. Wales/
26. United Kingdom.ti,ab.
27. United Kingdom/
28. UK.ti,ab.
29. 1 OR 2 OR 4 OR 4 OR 5
30. 6 OR 7 OR 8 OR 9 OR 10 OR 11 OR 12 OR 13 OR 14 OR 15 OR 16 OR 17
31. 18 OR 19 OR 20 OR 21 OR 22 OR 23 OR 24 OR 25 OR 26 OR 27 OR 28
32. 29 AND 30 AND 31

For peer review only

Medline (Ovid MEDLINE® 1946 to present)

1. Physical activity.ti,ab.
2. Exercise.ti,ab.
3. Exercise/
4. Sedentary behaviour.ti,ab.
5. Sedentary behaviour/
6. Having a child.ti,ab.
7. Having children.ti,ab.
8. Childbearing.ti,ab.
9. Preconception.ti,ab.
10. Pregnancy.ti,ab.
11. Pregnancy/
12. Postpartum.ti,ab.
13. Post-partum.ti,ab.
14. Parenthood.ti,ab.
15. Motherhood.ti,ab.
16. Fatherhood.ti,ab.
17. England.ti,ab.
18. England/
19. Northern Ireland.ti,ab.
20. Northern Ireland/
21. Scotland.ti,ab.
22. Scotland/
23. Wales.ti,ab.
24. Wales/
25. United Kingdom.ti,ab.
26. United Kingdom/
27. UK.ti,ab.
28. 1 OR 2 OR 3 OR O4 OR 5
29. 6 OR 7 OR 8 OR 9 OR 10 OR 11 OR 12 OR 13 OR 14 OR 15 OR 16
30. 17 OR 18 OR 19 OR 20 OR 21 OR 22 OR 23 OR 24 OR 25 OR 26 OR 27
31. 28 AND 29 AND 30

PsycInfo (APA PsychInfo 1806 to January Week 5 2022)

1. Physical activity.ti,ab.
2. Physical activity/
3. Exercise.ti,ab.
4. Exercise/
5. Sedentary behaviour.ti,ab.
6. Sedentary behaviour/
7. Having a child.ti,ab.
8. Having children.ti,ab.
9. Childbearing.ti,ab.
10. Preconception.ti,ab.
11. Pregnancy.ti,ab.
12. Pregnancy/
13. Postpartum.ti,ab.
14. Post-partum.ti,ab.
15. Parenthood.ti,ab.
16. Motherhood.ti,ab.
17. Fatherhood.ti,ab.
18. England.ti,ab.

For peer review only

19. Northern Ireland.ti,ab.
20. Scotland.ti,ab.
21. Wales.ti,ab.
22. United Kingdom.ti,ab.
23. UK.ti,ab.
24. 1 or 2 or 3 or 4 or 5 or 6
25. 7 or 8 or 9 or 10 or 11 or 12 or 13 or 14
26. 15 or 16 or 17 or 18 or 19 or 20
27. 21 and 22 and 23

Web of Science (Web of Science Core Collection)
In title:

((TI=("physical activity" OR exercise OR “sedentary behaviour”)) AND TI=("having a child" OR "having children" OR childbearing OR preconception OR pregnancy OR postpartum OR post-partum OR parenthood OR motherhood OR fatherhood)) AND TI=(england OR "northern ireland" OR scotland OR wales OR "united kingdom" OR uk)

In abstract:

((AB=("physical activity" OR exercise OR “sedentary behaviour”)) AND AB=("having a child" OR "having children" OR childbearing OR preconception OR pregnancy OR postpartum OR post-partum OR parenthood OR motherhood OR fatherhood)) AND AB=(england OR "northern ireland" OR scotland OR wales OR "united kingdom" OR uk)

GREY LITERATURE SEARCH STRATEGIES

Google Scholar
allintitle: ("physical activity" OR exercise OR “sedentary behaviour”) AND ("having a child" OR "having children" OR childbearing OR preconception OR pregnancy OR postpartum OR post-partum OR parenthood OR motherhood OR fatherhood) AND england

allintitle:("physical activity" OR exercise OR “sedentary behaviour”), ("having a child" OR "having children" OR childbearing OR preconception OR pregnancy OR postpartum OR post-partum OR parenthood OR motherhood OR fatherhood) AND "northern ireland"

allintitle: ("physical activity" OR exercise OR “sedentary behaviour”) AND ("having a child" OR "having children" OR childbearing OR preconception OR pregnancy OR postpartum OR post-partum OR parenthood OR motherhood OR fatherhood) AND Scotland

allintitle: ("physical activity" OR exercise OR “sedentary behaviour”) AND ("having a child" OR "having children" OR childbearing OR preconception OR pregnancy OR postpartum OR post-partum OR parenthood OR motherhood OR fatherhood) AND wales

allintitle: ("physical activity" OR exercise OR “sedentary behaviour”) AND ("having a child" OR "having children" OR childbearing OR preconception OR pregnancy OR postpartum OR post-partum OR parenthood OR motherhood OR fatherhood) AND "united kingdom"

allintitle: ("physical activity" OR exercise OR “sedentary behaviour”) AND ("having a child" OR "having children" OR childbearing OR preconception OR pregnancy OR postpartum OR post-partum OR parenthood OR motherhood OR fatherhood) AND uk

ProQuest
In title:

ti("physical activity" OR exercise OR “sedentary behaviour”) AND ti("having a child" OR "having children" OR childbearing OR preconception OR pregnancy OR postpartum OR post-partum OR

For peer review only

parenthood OR motherhood OR fatherhood) AND ti(england OR "northern ireland" OR scotland OR wales OR "united kingdom" OR uk)

In abstract:

ab("physical activity" OR exercise OR “sedentary behaviour”) AND ab("having a child" OR "having children" OR childbearing OR preconception OR pregnancy OR postpartum OR post-partum OR parenthood OR motherhood OR fatherhood) AND ab(england OR "northern ireland" OR scotland OR wales OR "united kingdom" OR uk)

Websites
For example, “Best Beginning’s” and “Tommy’s”.

For peer review only
